# The global burden and trend of Clostridioides difficile and its association with world antibiotic consumption, 1990–2019

**DOI:** 10.7189/jogh.14.04135

**Published:** 2024-08-16

**Authors:** Yonghao Chen, Xiaoxi Xie, Qintao Ge, Xiaogang He, Zhiyuan Sun, Yanni Li, Yaoyu Guo, Chong Geng, Xiao Li, Chunhui Wang

**Affiliations:** 1 Department of Gastroenterology and Hepatology, West China Hospital of Sichuan University, Chengdu, Sichuan, PR China; 2 Department of Urology, The First Affiliated Hospital of Anhui Medical University, Hefei, Anhui, PR China; 3 Translational Medical Research, Orthopaedics Department, Medical Faculty Mannheim, Heidelberg University, Mannheim, Germany; 4 School of Computer Science and Technology, University of Science and Technology of China, Hefei, Anhui, PR China; 5 Centre of Pancreatitis, West China Hospital of Sichuan University, Chengdu, Sichuan, PR China

## Abstract

**Background:**

To estimate the global trends and disease burden of Clostridioides difficile infection (CDI) and its correlation with worldwide antibiotic consumption.

**Methods:**

Clostridioides difficile infection and antibiotic consumption data were retrieved from the Global Burden of Disease 2019, ResistanceMap-AntibiocUse, Food and Drug Administration (FDA) Adverse Event Reporting System, and Global Antimicrobial Resistance and Use Surveillance System. Jointpoint regression and age-period-cohort model were developed to show the global trends and burden of CDI. Correlation tests were calculated to explore the relationship between CDI and antibiotics.

**Results:**

Globally, CDI is the most significant one with a high-rocketing burden increase rate among 13 pathogens causing diarrheal deaths and disability-adjusted life years (DALYs). The age-standardised death rate (ASDR) increased from 0.19 in 1990 to 0.43 in 2019, in which the elderly and females are at higher risk. A rapid increase in ASDR in high to middle sociodemographic index (SDI) regions such as North America (average annual percentage change (AAPC) = 7.71%), Andean (AAPC = 7.82%), and Southern Latin America (AAPC = 11.08%) was identified. Antibiotic consumption has a significant positive correlation with CDI with different risk stratifications.

**Conclusions:**

The global burden of CDI has continuously increased for the past 30 years, especially in high to middle-SDI regions. World antibiotic consumption showed a strong positive correlation with CDI with different risk stratification. More effective prevention and control measures should be implemented in these critical regions, with a specific emphasis on vulnerable populations, to mitigate the spread of epidemics.

Clostridioides difficile, as a spore-forming, gram-positive bacillus responsible for the spectrum from infectious diarrhoea and pseudomembranous colitis to megacolon and death, stands as one of the most common health care-associated infections [1,2]. Clostridioides difficile’s spores can easily colonise in intestinal epithelia and activate when antibiotics disrupt healthy indigenous microbiota, making it the leading cause of antibiotic-associated diarrhoea. By producing TcdA (toxin A) and TcdB (toxin B), toxigenic strains depolymerise epithelia cells’ actin through Rho GTPase inactivation and then stimulate the inflammatory cascade, leading to gut damage and severe diarrhoea [1,3]. The rapid spreading of hypervirulent strains such as North American pulsed-field gel electrophoresis type 1 (NAP1)/027 propels Clostridioides difficile infection (CDI) to become an urgent threat infection announced by the Centres for Disease Control and Prevention (CDC) [4]. The report published in 2015 based on CDC Emerging Infections Program (EIP) data in 2011 revealed 453 000 incident infections and 29 000 deaths in the USA [5]. It has been demonstrated from CDC’s Antibiotic Resistance Threats in the USA that CDI caused around 223 900 estimated cases in hospitalised patients in 2017, and 12 800 estimated deaths, and 1 billion US dollars (USD) attributable health care costs [6]. A European prospective point-prevalence multicentre study reported 7.0 cases of CDI per 10 000 patient-bed days in hospital [7]. And some studies reported considerably varied mortalities of CDI: the all-cause mortality at 30-day is from 9 to 38%, and attributable mortality at 30 days could range from 5.7 to 6.9% [8], indicating the importance of understanding the epidemiology characteristics of CDI.

Instead of analysing from a global vision, previous studies about the burden and trends of CDI are limited in certain regions and periods and have high heterogeneity due to wide-ranging approaches to testing and analysing. To explore the global burden and trends attributed to CDI from a planet prospect, open data from the Global Burden of Disease 2019 Study (GBD 2019) were retrieved. As a comprehensive picture of mortality and disability across countries, time, age, and sex, GBD 2019 could help us determine CDI's cross-region magnitude and scope under a standardised level [9]. Moreover, the worldwide antibiotic consumption data were collected from the ResistanceMap-AntibiocUse map [10] and Global Antimicrobial Resistance and Use Surveillance System (GLASS) to estimate the correlation between the burden of CDI and antibiotic usage and guide priority for prevention, cross-referencing with CDI in FDA Adverse Event Reporting System (FAERS) [11]. In this study we set three major objectives to fill the knowledge gaps: 1) to provide a comprehensive overview of the global disease trends and burden of death and disability-adjusted life years (DALYs) caused by CDI over the past three decades in different regions; 2) to examine the age, period, and cohort effects on CDI burden and how they differ between sexes and age groups; 3) to explore the association between antibiotic consumption patterns and the burden of CDI at the global level and investigate the risk stratification of different antibiotics.

## METHODS

Detailed data collection, data washing, statistical methods, and parameter explanations are included in Methods S1–5 in the **Online Supplementary Document**.

### Data sources

Data on the global burden of CDI were obtained from the Global Burden of Disease Study 2019 using the open online GHDx query tool [12]. Informed consent for accessing the GBD data was waived by the University of Washington Institutional Review Board [13]. The global antibiotic consumption data were obtained from ResistanceMap-AntibiocUse developed by OneHealthTrust (Centre for Disease Dynamics, Economics & Policy) [10] and Global Antimicrobial Resistance and Use Surveillance System (GLASS, https://www.who.int/initiatives/glass). The antibiotic adverse event data were collected from the publicly available FAERS database from 2004 to 2023, containing drug-associated post-market adverse events reported by manufacturers, patients, or health care professionals at the point of care [11]. Age-standardised death rates attributed to unsafe water, lack of hand-washing facilities, poor hand hygiene, low coverage of health service, low hospital beds, and a low number of specialised doctors and nurses were also retrieved from GBD 2019 and Wordl Health Organization data sets for confounding variables analysis.

### Statistical analysis

The Jointpoint regression, also called change point regression, was applied to access annual percentage changes (APCs) and average annual percentage changes (AAPCs) in time series data [14]. Age-period-cohort models were used to estimate period, age, and cohort effects in temporal trends, referring to the differences across age groups, birth years, and other people-related risk factors [15]. Estimated annual percentage changes (EAPCs) were manually calculated to measure the annual trend changes in death and DALY rates. Spearman correlation coefficients (ρ) were calculated to evaluate the relationship between different antibiotic consumption and age-standardised death rates (ASDR). The Reporting Odds Ratios (ROR) were calculated to estimate the association between CDI and individual antibiotics. *P*-value <0.05 was considered statistically significant. (Methods in the **Online Supplementary Document**). All statistical analyses were performed by R software (version 4.3.1, Vienna, Austria, 2023).

## RESULTS

### Global burden and trends of CDI with age-period-cohort effects

Globally, the number of deaths due to CDI infection increased dramatically from 8321 (95% uncertainty interval (UI) = 6637, 10 469) in 1990 to 32 134 (95% UI = 28 131, 36 549) in 2019. The outcome showed an increase of ASDRs from 0.19 (95% UI = 0.16, 0.23) in 1990 to 0.43 (95% UI = 0.37, 0.49) in 2019 (AAPC = 2.79%, *P* < 0.05) (**Table 1**). The annual rates of change in deaths percent and DALYs number were 641.2 and 1.03 respectively, making CDI the most significant one with a high-rocketing burden increase rate among 13 pathogens causing diarrheal deaths and DALYs included in GBD 2019 (Tables S1–2 in the **Online Supplementary Document**).

**Table 1:** The death numbers and ASDR of Clostridioides difficile in 1990 and 2019 with EAPCs and AAPCs

Variables	1990		2019			
	**Death number (95% UI)**	**ASDR (95% UI)**	**Death number (95% UI)**	**ASDR (95% UI)**	**EAPC (95% CI)**	**AAPC, % (95% CI)**
Global	8321 (6637, 10 469)	0.19 (0.16, 0.23)	32 134 (28 131, 36 549)	0.43 (0.37, 0.49)	3.70 (3.11, 4.30)	2.79* (2.66, 2.93)
Gender						
*Male*	4194 (3263, 5406)	0.20 (0.17, 0.24)	14 800 (12 892, 16 810)	0.45 (0.39, 0.52)	3.70 (3.16, 4.24)	2.81* (2.66, 2.96)
*Female*	4127 (3371, 5113)	0.18 (0.15, 0.22)	17 333 (14 873, 19 960)	0.41 (0.35, 0.48)	3.80 (3.16, 4.45)	2.83* (2.67, 2.98)
SDI rank						
*High SDI*	2914 (2586, 3249)	0.30 (0.27, 0.33)	20 608 (17 624, 23 649)	0.99 (0.87, 1.11)	5.43 (4.59, 6.28)	4.26* (3.98, 4.55)
*High-middle SDI*	1781 (1361, 2271)	0.17 (0.13, 0.22)	4842 (4101, 5665)	0.30 (0.25, 0.35)	2.30 (2.14, 2.47)	1.88* (1.64, 2.12)
*Middle SDI*	2521 (1737, 3553)	0.14 (0.10, 0.19)	4921 (3722, 6306)	0.22 (0.17, 0.28)	1.58 (1.47, 1.69)	1.45* (1.33, 1.57)
*Low-middle SDI*	775 (477, 1247)	0.06 (0.04, 0.09)	1326 (847, 1956)	0.08 (0.05, 0.11)	0.88 (0.64, 1.13)	0.88* (0.56, 1.21)
*Low SDI*	326 (195, 548)	0.05 (0.03, 0.08)	424 (221, 766)	0.03 (0.02, 0.06)	−1.67 (−1.89, −1.45)	−1.38* (−1.64, −1.11)
GBD region						
*Andean Latin America*	11 (5, 20)	0.03 (0.01, 0.05)	160 (109, 227)	0.26 (0.18, 0.37)	8.14 (7.98, 8.31)	7.82* (6.72, 8.93)
*Australasia*	43 (38, 48)	0.20 (0.18, 0.23)	243 (203, 284)	0.46 (0.39, 0.52)	3.37 (3.03, 3.72)	2.95* (2.58, 3.33)
*Caribbean*	29 (21, 37)	0.09 (0.07, 0.11)	86 (67, 109)	0.18 (0.14, 0.22)	2.61 (2.47, 2.74)	2.50* (1.90, 3.10)
*Central Asia*	26 (14, 45)	0.04 (0.02, 0.06)	47 (30, 69)	0.05 (0.03, 0.08)	1.49 (1.32, 1.67)	1.42* (0.81, 2.03)
*Central Europe*	59 (44, 76)	0.05 (0.04, 0.06)	202 (148-268)	0.12 (0.10, 0.15)	3.98 (3.63, 4.34)	3.35* (3.11, 3.60)
*Central Latin America*	160 (96, 263)	0.09 (0.05, 0.13)	933 (681, 1239)	0.41 (0.29, 0.54)	5.51 (5.31, 5.70)	5.52* (4.82, 6.21)
*Central sub-Saharan Africa*	31 (16, 56)	0.05 (0.03, 0.08)	71 (39, 121)	0.05 (0.03, 0.08)	−0.11 (−0.38, 0.15)	−0.13 (−0.34, −0.09)
*East Asia*	906 (605, 1293)	0.08 (0.05, 0.11)	1927 (1442, 2633)	0.13 (0.10, 0.17)	1.63 (1.30, 1.97)	1.68* (1.30, 2.07)
*Eastern Europe*	129 (81, 184)	0.06 (0.04, 0.09)	211 (159, 268)	0.09 (0.07, 0.11)	1.45 (1.14, 1.76)	1.51* (0.44, 2.59)
*Eastern sub-Saharan Africa*	125 (68, 219)	0.05 (0.03, 0.05)	283 (164, 486)	0.06 (0.04, 0.10)	0.42 (0.30, 0.53)	0.37* (0.19, 0.55)
*High-income Asia Pacific*	634 (518, 762)	0.37 (0.30, 0.44)	2650 (2066, 3277)	0.54 (0.44, 0.64)	1.33 (1.16, 1.5)	1.30* (0.89, 1.71)
*High-income North America*	707 (643, 749)	0.21 (0.19, 0.22)	11 175 (9833, 12 402)	1.68 (1.51, 1.84)	9.21 (7.68, 10.76)	7.71* (7.11, 8.3)
*North Africa and Middle East*	98 (50, 177)	0.02 (0.01, 0.04)	395 (272, 548)	0.07 (0.05, 0.10)	3.48 (3.40, 3.56)	3.7* (3.23, 4.17)
*Oceania*	9 (7, 13)	0.15 (0.11, 0.19)	6 (4, 9)	0.05 (0.03, 0.07)	−3.83 (−3.90, −3.77)	−3.84* (−3.97, −3.71)
*South Asia*	846 (501, 1365)	0.07 (0.04, 0.10)	1311 (808, 1978)	0.08 (0.05, 0.11)	−0.07 (−0.24, 0.11)	0.27 (−0.15, 0.69)
*Southeast Asia*	1053 (732, 1459)	0.22 (0.15, 0.29)	1387 (982, 1859)	0.22 (0.16, 0.29)	−0.13 (−0.24, −0.01)	−0.03 (−0.23, 0.17)
*Southern Latin America*	14 (8, 22)	0.03 (0.02, 0.05)	477 (356, 621)	0.59 (0.44, 0.75)	11.55 (10.71-12.39)	11.08* (10.38, 11.79)
*Southern sub-Saharan Africa*	457 (339, 595)	0.83 (0.63, 1.06)	703 (531, 902)	0.88 (0.67, 1.12)	0.54 (0.26, 0.83)	0.22 (−0.19, 0.64)
*Tropical Latin America*	534 (359, 751)	0.31 (0.21, 0.44)	556 (404, 778)	0.27 (0.20, 0.37)	0.08 (−0.67, 0.83)	−0.43* (−0.69, −0.17)
*Western Europe*	1700 (1474, 1919)	0.30 (0.27, 0.34)	8389 (6900, 9955)	0.78 (0.66, 0.90)	4.36 (3.83, 4.89)	3.24* (2.95, 3.52)
*Western sub-Saharan Africa*	750 (540, 1036)	0.35 (0.26, 0.48)	922 (620, 1320)	0.18 (0.12, 0.24)	−2.82 (−3.09, −2.55)	−2.69* (−2.98, −2.4)

AAPC – average annual percentage change, ASDR – age-standardised death rate, CI – confidence interval, EAPC – estimated annual percentage changes, GBD – global burden of disease, SDI – sociodemographic index, UI – uncertainty interval

*Statistically significant (*P* < 0.05).

**Figure 1:**
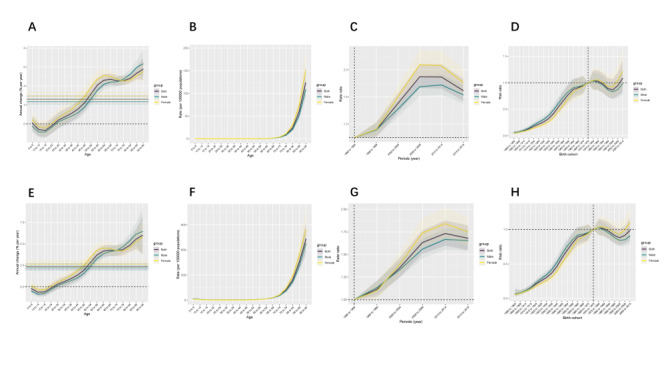
Age-period-cohort analysis of ASDR and ASRD of CDI. **Panel A**. Local drifts with net drifts for CDI-caused deaths. **Panel B**. Longitudinal age curves for CDI-caused deaths. **Panel C**. Period effects on deaths. **Panel D**. Birth cohort effects on deaths. **Panel E**. Local drifts with net drifts for CDI-caused DALYs. **Panel F**. Longitudinal age curves for CDI-caused DALYs. **Panel G**. Period effects on DALYs. **Panel H**. Birth cohort effects on DALYs. ASDR – age-standardised death rate, ASRD – age-standardised rates of disability-adjusted life years, CDI – Clostridioides difficile infection, DALY – disability-adjusted life years

**Figure 1**, panels A, E illustrate the change of percentage per year of local drifts with net drifts for CDI-caused deaths (2.5, 95% confidence interval (CI) = 1.75, 3.19) and DALYs (2.4, 95% CI = 1.96, 2.79). The global trends in CDI-caused deaths and DALYs are increasing from 1990 to 2019. The deaths overall net drifts for females during the whole period are higher than those in the male group (deaths = 2.76, 95% CI = 1.96, 3.56 vs. 2.3, 95% CI = 1.65, 2.95), with slightly higher AAPC in females ASDR (2.83, 95% CI = 2.67, 2.98 vs. 2.81, 95% CI = 2.66, 2.96) and age-standardised rates of DALYs (ASRD) (1.50, 95% CI = 1.33, 1.67 vs. 1.31, 95% CI = 1.09, 1.54) (**Table 1**, **Table 2**).

**Table 2:** The DALY numbers and ASRD of Clostridioides difficile in 1990 and 2019 with EAPCs and AAPCs

Variables	1990		2019			
	DALYs No.*10^3^ (95% CI)	**Age-standardised DALYs rate per 100 000 No. (95% CI)**	DALYs No.*10^3^ (95% CI)	**Age-standardised DALYs rate per 100 000 No. (95% CI)**	**EAPC (95% CI)**	**AAPC, % (95% CI)**
Global	428.56 (306.02, 605.83)	7.79 (5.78, 10.66)	870.81 (722.99, 1052.3)	11.69 (9.62, 14.25)	1.72 (1.46, 1.98)	1.39* (1.23, 1.55)
Gender						
*Male*	233.56 (165.58, 330.20)	8.43 (6.27, 11.55)	446.22 (367.32, 546.69)	12.36 (10.18, 15.20)	1.57 (1.34, 1.81)	1.31* (1.09, 1.54)
*Female*	195.00 (139.60, 274.35)	7.11 (5.26, 9.81)	424.59 (350.86, 516.16)	11.06 (8.96, 13.73)	1.93 (1.64, 2.22)	1.50* (1.33, 1.67)
SDI rank						
*High SDI*	68.76 (62.43, 75.46)	8.29 (7.40, 9.26)	314.09 (284.81, 341.44)	18.86 (17.46, 20.24)	4.32 (3.15, 5.51)	2.84* (2.64, 3.04)
*High-middle SDI*	101.83 (72.53, 140.02)	9.25 (6.63, 12.79)	142.58 (118.26, 173.28)	11.27 (9.10, 14.15)	0.33 (0.02, 0.64)	0.58* (0.41, 0.75)
*Middle SDI*	176.40 (117.86, 260.70)	9.10 (6.17, 13.24)	293.82 (215.66, 389.04)	13.83 (10.08, 18.43)	1.26 (0.93, 1.60)	1.41* (1.13, 1.68)
*Low-middle SDI*	57.19 (34.93, 95.15)	3.97 (2.43, 6.38)	89.74 (56.49, 140.28)	4.97 (3.13, 7.70)	0.54 (−0.15, 1.22)	0.75* (0.34, 1.15)
*Low SDI*	24.15 (14.07, 42.35)	3.36 (2.03, 5.56)	30.21 (15.11, 57.01)	2.13 (1.11, 3.83)	−1.89 (−2.47, 1.30)	−1.59* (−1.88, −1.31)
GBD region						
*Andean Latin America*	0.77 (0.34, 1.50)	1.65 (0.77, 3.11)	10.15 (6.46, 15.42)	15.99 (10.18, 24.21)	8.14 (7.96-, 8.33)	7.91* (7.37, 8.45)
*Australasia*	0.93 (0.83, 1.03)	4.55 (4.03, 5.12)	3.59 (3.18, 4.02)	8.76 (7.79, 9.85)	2.66 (2.34, 2.98)	2.35* (2.02, 2.69)
*Caribbean*	1.66 (1.17, 2.29)	4.50 (3.23, 6.01)	3.53 (2.72, 4.59)	7.71 (5.92, 10.10)	1.93 (1.8, 2.05)	1.79* (1.18, 2.41)
*Central Asia*	1.75 (0.90, 3.22)	2.23 (1.17, 3.94)	2.98 (1.81, 4.62)	3.15 (1.93, 4.84)	1.32 (1.09, 1.55)	1.27* (0.60, 1.95)
*Central Europe*	2.84 (2.14, 3.71)	2.56 (1.87, 3.38)	5.89 (4.71, 7.27)	5.08 (4.18, 6.09)	3.12 (2.67, 3.57)	2.31* (1.86, 2.77)
*Central Latin America*	11.86 (6.77, 20.21)	5.73 (3.42, 9.54)	66.34 (47.39, 88.77)	29.19 (20.78, 39.45)	5.57 (5.37, 5.77)	5.71* (5.09, 6.34)
*Central sub-Saharan Africa*	2.30 (1.09, 4.37)	3.04 (1.57, 5.36)	5.15 (2.76, 9.11)	3.03(1.69, 5.18)	−0.09 (−0.39, 0.20)	−0.11 (−0.36, 0.14)
*East Asia*	57.93 (36.95, 88.56)	4.76 (3.07, 7.29)	78.32 (59.00, 107.39)	6.50 (4.95, 8.65)	0.87 (0.44, 1.3)	1.07* (0.67, 1.47)
*Eastern Europe*	7.59 (4.76, 10.99)	3.75 (2.35, 5.53)	8.17 (6.36, 10.04)	4.35 (3.41, 5.38)	0.52 (0.24, 0.79)	0.65 (−0.50, 1.82)
*Eastern sub-Saharan Africa*	9.39 (5.10, 17.14)	3.52 (1.97, 6.03)	20.43 (11.52, 36.34)	3.90 (2.29, 6.58)	0.27 (0.14, 0.4)	0.21* (0.00, 0.42)
*High-income Asia Pacific*	18.04 (15.70, 20.57)	11.38 (9.82, 13.14)	40.11 (33.08, 47.61)	12.95 (11.21, 15.09)	0.49 (0.35, 0.63)	0.41* (0.13, 0.7)
*High-income North America*	16.77 (14.74, 18.55)	5.90 (5.02, 6.67)	175.68 (162.34, 187.67)	30.08 (28.12, 31.90)	7.23 (6.13, 8.35)	5.83* (5.49, 6.18)
*North Africa and Middle East*	7.17 (3.52, 13.48)	1.61 (0.82, 2.91)	23.69 (15.73, 34.88)	3.88 (2.60, 5.65)	2.78 (2.69, 2.87)	3.03* (2.47, 3.60)
*Oceania*	0.63 (0.44, 0.92)	8.39 (6.01, 11.57)	0.38 (0.22, 0.64)	2.59 (1.54, 4.16)	−4.00 (−4.07, −3.93)	−3.99* (−4.17, −3.81)
*South Asia*	80.48 (47.65, 128.45)	4.46 (2.63, 7.19)	60.45 (34.98, 102.11)	4.40 (2.64, 7.02)	−0.45 (−0.61, −0.29)	−0.06 (−0.53, 0.42)
*Southeast Asia*	71.99 (49.11, 105.34)	13.40 (9.28, 18.88)	79.82 (55.51, 110.41)	12.45 (8.71, 17.41)	−0.43 (−0.54, −0.32)	−0.28 (−0.60, 0.03)
*Southern Latin America*	0.76 (0.40, 1.34)	1.51 (0.81, 2.65)	11.41 (9.23, 13.81)	15.65 (12.83, 18.88)	8.85 (8.29, 9.42)	8.51* (8.03, 9.00)
*Southern sub-Saharan Africa*	31.06 (22.66, 41.86)	51.08 (37.56, 67.15)	44.09 (33.15, 57.58)	53.31 (40.08, 69.37)	0.39 (0.1, 0.68)	0.22 (−0.14, 0.59)
*Tropical Latin America*	39.95 (26.59, 57.43)	22.38 (15.09, 31.92)	31.47 (22.54, 42.81)	16.48 (11.73, 22.85)	−0.73 (−1.52, 0.08)	−1.02* (−1.48, −0.56)
*Western Europe*	31.43 (28.64, 34.13)	6.96 (6.22, 7.81)	112.47 (98.98, 126.64)	13.72 (12.51, 14.98)	3.33 (2.79, 3.88)	2.29* (1.98, 2.60)
*Western sub-Saharan Africa*	53.29 (37.52, 75.67)	21.61 (15.63, 29.52)	66.69 (44.51, 98.42)	11.41 (7.67, 16.33)	−2.68 (−2.95, −2.41)	−2.55* (−2.87, −2.23)

AAPC – average annual percentage change, ASRD – age-standardised rates of DALYs, CI – confidence interval, DALY – disability-adjusted life years, EAPC – estimated annual percentage changes, GBD – global burden of disease, SDI – sociodemographic index, UI – uncertainty interval

*Statistically significant (*P* < 0.05).

The exponentially distributed longitudinal age curves in **Figure 1**, panels B, F show the prominent increases in deaths and DALYs after 67.5 years old, with the steepest increases over 87.5 years old. The increments of deaths and DALYs in the male group slightly lagged behind those in the female group. Patients over 92.5 years old have the highest rate of deaths (67.9, 95% CI = 46.4, 99.5) and DALYs (449.1, 95% CI = 333.9, 604.1).

After controlling the age and cohort effects, period effects on deaths and DALYs were illustrated in **Figure 1**, panels C, G. Both the deaths and DALYs show an overall increasing trend from 1990 to 2009 (Relative risk (RR) of 2002 = 1), thereafter a plateau from 2009 to 2014 and slightly decreased after 2014. From 1990 to 2019, the RR for deaths increased from 0.76 (95% CI = 0.63, 0.92) to 1.26 (95% CI = 1.09, −1.47). The absolute values and increasing rates for RR in females are consistently higher than those in males. Birth cohort effects on deaths and DALYs (**Figure 1**, panels D, H) illustrate similar consistent increasing trends in men and women. From 1900 to 2010, the RR of deaths rose from 0.10 (95% CI = 0.04, 0.21) to 1.31 (95% CI = 0.71, 2.44).

### Sociodemographic index (SDI)-quintile level and regional specific of CDI-associated deaths and DALYs

**Figure 2:**
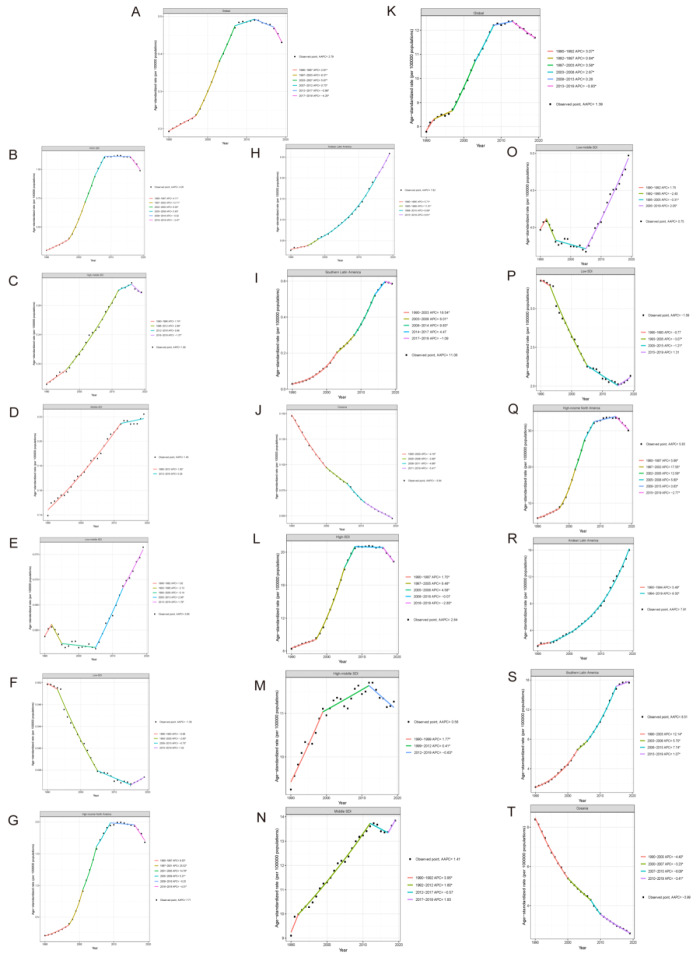
Jointpoint analysis based on SDI-quintile level and regional specific of CDI associated deaths and DALYs. **Panel A**. Global CDI caused death trend. **Panel B**. High-SDI region CDI caused death trend. **Panel C**. High-middle SDI region CDI caused death trend. **Panel D**. Middle-SDI region CDI caused death trend. **Panel E**. Low-middle SDI region CDI caused death trend. **Panel F**. Low-SDI region CDI caused death trend. **Panel G**. High-income North America CDI caused death trend. **Panel H**. Andean Latin America CDI caused death trend. **Panel I**. Southern Latin America CDI caused death trend. **Panel J**. Oceania CDI caused death trend. **Panel K**. Global CDI caused DALYs trend. **Panel L**. High-SDI region CDI caused DALYs trend. **Panel M**. High-middle SDI region CDI caused DALYs trend. **Panel N**. Middle-SDI region CDI caused DALYs trend. **Panel O.** Low-middle SDI region CDI caused DALYs trend. **Panel P**. Low-SDI region CDI caused DALYs trend. **Panel Q**. High-income North America CDI caused DALYs trend. **Panel R.** Andean Latin America CDI caused DALYs trend. **Panel S**. Southern Latin America CDI caused DALYs trend. **Panel T**. Oceania CDI caused DALYs trend. CDI – Clostridioides difficile infection, DALY – disability-adjusted life years, SDI – sociodemographic index

Over the past 30 years, the deaths and DALYs present significant growth trends among high-, high-middle-, middle- and low-middle SDI regions, but a downtrend in low SDI regions (**Table 1**, **Table 2**). The high SDI region bore the heaviest burden from CDI-caused deaths and DALYs and the most significant AAPC. The death number in high SDI regions high-rocketed from 2941 (95% UI = 2586, 3249) in 1990 to 20 608 (95% UI = 17 624, 23 649) in 2019, with an AAPC of 4.26 (95% CI = 3.98, 4.55; *P* < 0.05). Jointpoint analysis indicates similar changes model of ASDR and ASRD in the high-SDI region and global: rising-plateau-slight decreasing (**Figure 2**, panels A, B, K, L). With the downgrading of SDI, the burdens of deaths and DALYs are also decreasing (ASDR-AAPC, high-SDI = 4.26, 95% CI = 3.98, 4.55; high-middle SDI = 1.88, 95% CI = 1.64, 2.12; middle-SDI = 1.45 (95% CI = 1.33, 1.57; low-middle SDI = 0.88, 95% CI = 0.56, 1.21; The trends of ASRD indicate similarity with ASDR). The change patterns of ASDR and ASRD in high-middle and middle regions are similar: rising-slight decreasing (**Figure 2**, panels C, D, M, N), while they present a plateau-lately increasing pattern in low-middle SDI region (**Figure 2**, panels E, O).

Zooming in for more specific regional burden exploration, CDI-caused regional deaths and DAYLs trends are similar to their respective superior SDI regions. As a member in the high-SDI region, high-income North America shows similarities with the latter in the rising-plateau-slight decreasing pattern (**Figure 2**, panels G, Q), and the change models of Andean Latin America (**Figure 2**, panels H, R) and Southern (**Figure 2**,panels I, S) are more similar to it in high-middle or middle SDI regions in a continuously increasing pattern, while the change models of Oceania (**Figure 2**, panel J, T) are similar to the trend of low-SDI region.

### The burden of CDI at the national level

The five largest death numbers are the United States of America, Germany, Japan, China, and India in 2019, with Germany replacing Nigeria in 1990 and becoming the top fifth country. From 1990 to 2019, the annual rate of change in ASDR sharply increased in Latin American countries or territories: Argentina (29.75, 95% CI = 13.98, 59.30), Paraguay (21.85, 95% CI = 10.35, 49.0), Uruguay (18.89, 95% CI = 11.58, 31.11); European countries or territories: Austria (13.07, 95% CI = 10.79, 15.77), Colombia (12.62, 95% CI = 6.59, 24.15), North American countries or territories: USA (7.36, 95% CI = 6.54, 8.17), Canada (5.42, 95% CI = 4.30, 6.69), Asia countries or territories: Saudi Arabia (9.20, 95% CI = 4.68, 20.34), Oman (9.00, 95% CI = 4.52, 18.92). The annual rate change of ASDR in most countries or territories presents an upward trend (Table S3 in the Online **Supplementary Document**).

### Associations between regional CDI burden and antibiotics consumption

**Figure 3:**
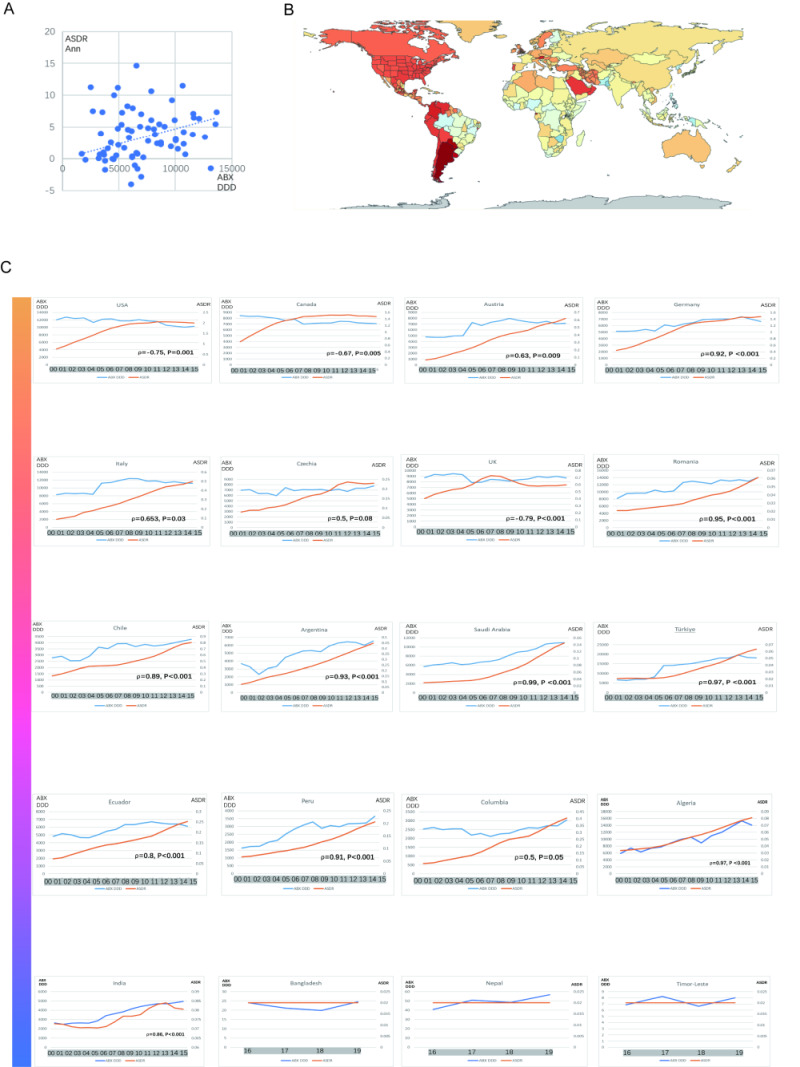
Associations between Regional CDI burden and antibiotics consumption. **Panel A**. Correlation between DDDs per 1000 inhabitants among 70 countries or territories and the annual change rate of ASDR. **Panel B**. Map of annual changes of ASDRs for both genders all around the world. **Panel C**. Correlation between DDDs per 1000 inhabitants and ASDRs in different SDI regions. CDI – Clostridioides difficile infection, DDD – number of defined daily dose, SDI – sociodemographic index

A rough correlation estimation (**Figure 3**, panel A) between 16 years the number of defined daily doses (DDDs) per 1000 inhabitants data among 70 countries or territories and the annual change rate of ASDR from 2000 to 2015 seems to present a positive correlation. The statistically significant correlation between the changes in age-standardised death rates (attributed to unsafe water, lack of hand-washing facilities, poor hand hygiene, low coverage of health service, low hospital beds, and a low number of specialised doctors or nurses) and annual changes of ASDR were not detected (*P* > 0.05, Figure S1 in the **Online Supplementary Document**).

Detailed analyses by countries and regions are potentially subdivided into different SDI levels to explicit comparisons to clarify variations in CDI trends across different SDIs (**Figure 3**, panels B, C). The USA, Canada, Austria, and Germany stand out as the four high-SDI countries with the highest annual rate of change in ASDR, spatially aggregating in the North American and Eurasia continents. The trends in antibiotic usage in the USA and Canada have shown a gradual decline in recent years following a period of high consumption. Similarly, the burden of CDI has also shown a deceleration in growth after years of rapid increase. Both trends have now stabilised, with a slight downward trajectory being observed. As an antibiotic-leading consumer in high-income North America, the USA’s DDDs per 1000 inhabitants consistently stay high, with a mild decrease after 2009. There was a rapid increase of ASDRs in the USA from 0.76 (95% CI = 0.70, 0.81) in 2000 to 2.05 (95% CI = 1.87, 2.19) in 2011, and then receded to 2.0 (95% CI = 1.82, 2.14) in 2015 together with falling DDDs, with a strong positive correlation between antibiotic consumption and ASDR (ρ = 0.75, *P* < 0.001). Elderly individuals over 70 are the main group with a significant fallen death rate after 2011 (death rate in 2011 = 30.49, 95% CI = 26.74, 33.60; in 2015 = 0.20, 95% CI = 24.91, 31.20). Canada has similar trends to the USA: DDDs per 1000 individuals are relatively stable at moderate-to-high levels but mildly decreasing from 2000 to 2015. Meanwhile, the ASDRs rapidly increased after 2000 (0.71, 95% CI = 0.61, 0.80) and then stabilised with a slight decrease after 2012 (ρ = 0.67, *P* = 0.005).

For high-SDI countries other than the USA and Canada and countries in high-middle, middle, and low-middle SDI areas except for the UK, the double curves of antibiotic consumption and CDI both presented a similar continuous rising pattern: high-SDI: Austria (ρ = 0.63, *P* = 0.009), Germany (ρ = 0.92, *P* < 0.001); high-middle SDI: Italy (ρ = 0.653, *P* = 0.03), Czechia (ρ = 0.5, *P* = 0.08), Romania (ρ = 0.95, *P* < 0.001); middle SDI: Chile (ρ = 0.89, *P* < 0.001), Argentina (ρ = 0.93, *P* < 0.001), Saudi Arabia (ρ = 0.99, *P* < 0.001), Turkey (ρ = 0.97, *P* < 0.001); low-middle SDI: Ecuador (ρ = 0.8, *P* < 0.001), Peru (ρ = 0.91, *P* < 0.001), Colombia (ρ = 0.5, *P* = 0.05), Algeria(ρ = 0.97, *P* < 0.001). The UK presents a waxing and waning but relatively stable antibiotic consumption data of around 8686 DDDs per 1000 inhabitants. After 2021, ASDRs caused by CDI in the UK gradually stabilised at around 0.60, after increasing year on year from 2000 to 2010 (ρ = –0.79, *P* < 0.001).

Countries in low-SDI regions with available data are most spatially aggregated in Africa and Oceania. The antibiotic consumption and CDI burden in India were both steady from 2000 to 2005 (DDDs per 1000 inhabitants: 2645 to 2857, ASDR = 0.072 to 0.073). From 2006 to 2013, the ASRD increased from 0.073 to 0.081 following a slight decrease from 2014 to 2015 to 0.081, with a meantime rapidly and continuously increasing antibiotic consumption from 3420 to 4950 DDDs per 1000 inhabitants. The CDI burden and trends for Bangladesh, Nepal, and Timor-Leste were fairly steady around 0.02 to 0.03 along with low antibiotic consumption with slight fluctuations, representing most low-SDI countries’ change patterns.

### Antibiotic risk stratification for Clostridioides difficile infection

**Figure 4:**
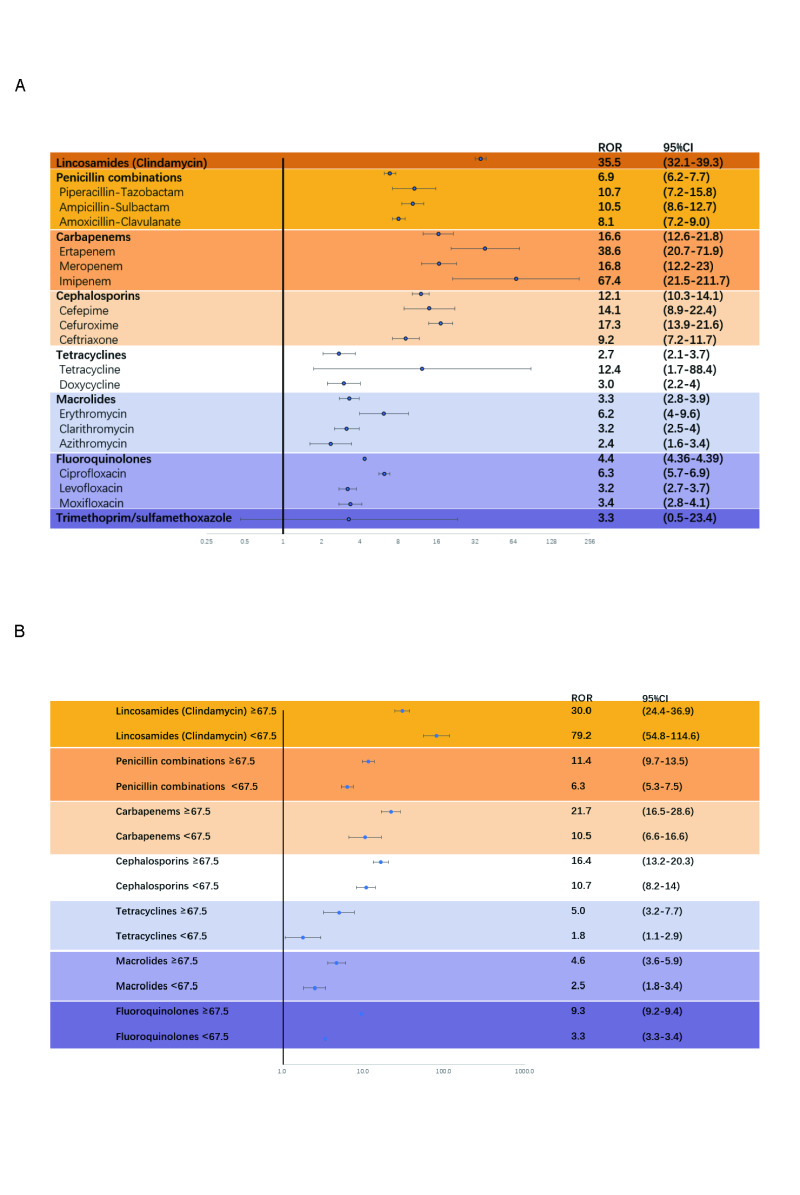
Antibiotic risk stratification. **Panel A**. Antibiotic risk stratification for Clostridioides difficile infection. **Panel B**. Subgroup analysis of antibiotic risk stratification for Clostridioides difficile infection in younger and older patients.

Focusing on countries with the most significant AAPC or EAPC (**Table 3**), DDDs per 1000 inhabitants of cephalosporins and fluoroquinolones are positively correlated with ASDRs in countries with the top 10 most significant annual change rates and available consumption data. And the pooled DDDs of broad-spectrum penicillins are positively correlated with seven countries, while those of narrow-spectrum penicillins and macrolides are positively correlated with nine countries. In the FAERS analysis (**Figure 4**, panel A), clindamycin has the highest ROR (35.5, 95% CI = 32.1, 39.3) among all included antibiotics, and carbapenems (ertapenem, meropenem, and imipenem) demonstrated the second highest CDI ROR (16.6, 95% CI = 12.6, 21.8). In contrast, trimethoprim-sulfamethoxazole has the lowest CDI ROR (3.3, 95% CI = 0.5, 23.4). Patients older than 67.5 who take penicillin, carbapenem, cephalosporins, tetracyclines, macrolides, and fluoroquinolones are at higher risk of CDI, while those younger than 67.5 who take clindamycin are under higher risk suffering from CDI (**Figure 4**, panel B).

**Table 3:** The association of different antibiotic consumption and Clostridioides difficile ASDR

	Correlation	Cephalosporins (DDD)	Fluoroquinolone (DDD)	Broad spectrum penicillin (DDD)	Narrow spectrum penicillin (DDD)	Macrolides (DDD)
Chile ASDR	ρ	0.89*	0.71*	0.96*	0.99*	0.91*
	*P*	0.00	0.00	0.00	0.00	0.00
Argentina ASDR	ρ	0.93*	0.91*	0.89*	0.93*	0.94*
	*P*	0.00	0.00	0.00	0.00	0.00
Uruguay ASDR	ρ	0.66*	0.99*	0.91*	0.87*	0.90*
	*P*	0.01	0.00	0.00	0.00	0.00
Ecuador ASDR	ρ	0.8*	0.99*	0.7*	0.91*	−0.74*
	*P*	0.00	0.00	0.00	0.00	0.00
Peru ASDR	ρ	0.98*	0.96*	0.56*	0.50*	0.89*
	*P*	0.00	0.00	0.02	0.05	0.00
Colombia ASDR	ρ	0.57*	0.57*	0.99*	1.00*	0.96*
	*P*	0.02	0.02	0.00	0.00	0.00
Austria ASDR	ρ	0.95*	0.50*	0.26	0.31	0.35
	*P*	0.00	0.05	0.34	0.24	0.19
USA ASDR	ρ	−0.89*	−0.84*	−0.1	−0.92*	−0.93*
	*P*	0.00	0.00	0.71	0.00	0.00
Saudi Arabia ASDR	ρ	0.89*	0.97*	0.99*	0.99*	−0.84*
	*P*	0.00	0.00	0.00	0.00	0.00
Portugal ASDR	ρ	0.61*	−0.85*	−0.43	−0.56*	−0.84*
	*P*	0.01	0.00	0.10	0.02	0.00

ASDR – age-standardised death rate, DDD – defined daily dose, ρ – Spearman correlation coefficient, *P* – P-value

*Statistically significant (*P* < 0.05).

## DISCUSSION

Although stands as the leading cause of antibiotic-associated diarrhoea, there is no research on its disease burden and epidemiological characteristics across different countries and territories. To our knowledge, this is the first study to utilise worldwide disease burden data for providing a comprehensive overview of the global disease trends and burden of death and DALYs caused by CDI over the past three decades from a planet vision and exploring the age, period, and cohort effects on CDI burden and how they differ between sexes and age groups. It is also the first world antibiotic consumption and FAERS database-based manuscript to explore the association between antibiotic consumption patterns and the burden of CDI at the global level and investigate the risk stratification of different antibiotics covering the longest years.

Globally, Clostridioides difficile is the most significant one with a high-rocketing burden increase rate among 13 pathogens causing diarrheal deaths and DALYs causing an astonishing 4-fold increase in death numbers. Significantly escalated ASDR and ASRD burden attributed to CDI should be regarded as a world major public health problem not only emphasised by medical professionals but all health-related policymakers.

Our global population analysis revealed women and elderly inhabitants over 67.5 years old have the most prominent increases in deaths and DALYs and the escalating rate increases with the growth of age. This agrees with the previous findings that advanced age is a well-characterised risk factor for CDI to increase 5 to 10-fold infection risk [16], and the RR for recurrence of CDI ranges from 1.01 to 1.04 for each additional year of age (1.3, 10.4 for elderly over 65 years old) [17], indicating the aging of populations is a prediction factor for treatment failure, ICU treatment, and poor outcomes [18]. Our outcomes of EAPC and AAPC both indicate a slightly higher increase in rates of ASDR and ASRD in women, in accordance with the earlier turnover point for women in age-period-cohort analysis outcome of deaths and DALYs. An increasing tendency over time for differences between sexes seems to be noticed, indicating the female group seems to be more easily to get injured younger than male: during the early years, peripartum women were promoted as an individual high-risk group prone to get worse or relapse [19].

Although the overall burden of CDI is rapidly increasing, the patterns of change vary across different regions, typically displaying a ‘chasing’ pattern that reflects regional disparities. High-burden areas of CDI are spatially concentrated in highly developed and moderately developed regions. For example, regions with advanced health care systems and high SDI such as the USA and Canada have experienced a rapid growth in CDI burden over nearly 25 years, but have seen a slowing growth trend with a slight decline in the past five years. Most other moderately to highly developed regions continue to show a persistent rise in CDI burden, with growth trends similar to those seen in the most developed areas over the previous 20 years. Less developed regions, after experiencing low CDI burdens for nearly 15 years, have only recently begun to see rapid increases, with curve patterns more akin to those seen at the onset in moderately developed regions. For low SDI areas, primarily represented by Pacific Island nations and countries in Africa, the burden of CDI is generally light and stable, with only minor fluctuations in decrease and increase. The global trend of CDI disease follows a pattern of stable low-level fluctuations to rapid growth, then to high-level stabilisation with slight declines, in line with economic development.

When it comes to the spreading risk factors of CDI, the antibiotic (dosage, type, applying time), hospitalisation (rate, coverage, sanitation), and epidemic ribotype should be taken into consideration.

Although the lack of significant correlation between changes in age-standardised death rates (attributed to unsafe water, lack of hand-washing facilities, poor hand hygiene, low coverage of health service, low hospital beds, and the low number of specialised doctors or nurses) and annual changes of ASDR could help us rule out some confounders, we were unable to further analyse the effects of the boundaries between health care-associated CDI (HA-CDI) or community-acquired CDI (CA-CDI) circulation and the epidemic strain type diversity due to the lack of global surveillance data, which were confounding factors regarded as important origins of epidemiology heterogeneity to explain the difference of statistical significance in global and regional antibiotic correlation analysis. In the past decade, the incidence of CA-CDI reached up to 30–40% of all patients [5,20] and England studies also showed that females account for 63–67% of CA-CDI cases, suggesting the wider community endemic trend. What is more, the CA-CDI is prone to influence younger who are less likely to be exposed to antibiotics: the 2021 Annual Report for the Emerging Infections Program for CDI from the CDC also indicates more CA-CDI than HA-CDI (55.9 vs. 54.3 cases per 100 000 persons), with an increasing incidence rate in women [21,22] indicating compounding effects of CA-CDI may be the reason for the increasing burden on women. Previous specific regions’ publications supported some outcomes in our analysis: in North America, the hypervirulent ribotype BI/NAP1/027 caused large epidemics from 2000 onwards in the USA and Canada with substantial morbidity and mortality [1]. In the USA, the number of hospitalisations with the diagnosis of CDI reached a plateau around 2009. There is a decrease in cases in patients over 65 years old and the HA-CDI number, announced by CDC’s Emerging Infections Program [23]. In our global analysis, after maintaining high-level antibiotic consumption for many years, the consumption of antibiotics slowly declined from 2009 in the USA, along with the decreases in ASDR and ASRD after 2011, especially for individuals over 70 years old. According to reports based on data from the Canadian Nosocomial Infections Surveillance Program (CNISP) network, the HA-CDI cases declined and stabilised in 2017 (3.85 per 10 000 patient days in 2017 vs. 6.03 in 2012) [24,25], in accordance with our deaths and DALYs outcome. The annual data of CDI from the UK Health Security Agency indicates a downward trend in annual counts and rates of CDI in the UK since 2007, along with a decreasing prevalence of BI/NAP1/027 [26,27]. However, the global hospitalisation rates and characteristics of patients hospitalised data sets were not established until the wide spread of coronavirus disease 2019 (COVID-19) in 2020, and we have not established a global surveillance network until now either. It will take a few more years to collect enough data for global trends estimation.

Hence, we finally focused on the major cause of CDI: the antibiotics. To capture the association between the antibiotic application and CDI, we chose the WHO-recognised indicator DDDs per 1000 inhabitants per day as the methodology to cover antibiotic types, drug use time, dose, hospitalisation time, infectious disease registration, and complications, allowing us to compare the antibiotic application from a global vision as we presented in the manuscript. Despite the extreme lack of data available for analysis in less developed regions, the WHO data from recent years indicate that, due to social development constraints, antibiotic use has remained at a consistently low level in these areas, several orders of magnitude lower than in highly developed regions. The infections of CDI in these areas typically fluctuate slightly around the mean value, with no significant changes observed in recent years. In some less developed regions (e.g. India), after years of minimal antibiotic use, antibiotic consumption has gradually begun to rise, and the CDI burden, which was previously stable at low levels, has also started to rise rapidly in the same years. In moderately developed regions, the majority of countries show very consistent annual increases in antibiotic consumption, and the burden of CDI similarly displays a pattern of rapid annual increases. In highly medically advanced countries like those in North America, after years of high and rising levels of antibiotic use concurrent with rapid increases in CDI, recent shifts in the mainstream medical community's recognition of the epidemiological risks posed by such scenarios have led to proactive control measures. Following the rapid rise in antibiotic consumption, recent antibiotic use has peaked and plateaued with a slight downward trend, and CDI has also notably shown a pattern change, reaching a plateau with a slight decline. The trends of antibiotic consumption and CDI in most countries and regions are highly correlated, and the patterns of antibiotic use closely align with the aforementioned CDI changes, indicating a strong global epidemiological link between antibiotic consumption and CDI changes.

Although we gradually focused on antibiotic consumption as the main cause following the logic of the Cause-and-Effect Analysis Chart, the global epidemic correlation analysis remains more of a correlation analysis and has weaker explanatory power for causality. We further selected the FAERS database for in-depth analysis, making it the first study to incorporate the broadest range of years in the risk stratification of antibiotics related to CDI. Previous specific year analyses and some meta-analysis results categorised CDI antibiotics into high-risk groups (clindamycin, cephalosporins, and fluoroquinolones) and medium-risk groups (penicillin and macrolides) [28–30]. The results of this large-scale, extensive data analysis confirm that the highest-risk antibiotic remains clindamycin, consistent with previous findings. The subsequent risk order of antibiotics shows slight differences from earlier studies such as carbapenems, cephalosporins, and penicillin. However, compared to previous studies, this research spans more years and includes more data, thus providing stronger support for the reliability of its results.

As the region that successfully slows down the increase of the burden of CDI, their strategies are worthy of emulation for rapidly increasing ones [31]. First, since inappropriate antibiotic use is the most important risk factor, stewardship as a successful effort to lower antibiotic consumption and slowdown the increase of CDI in high-income countries would also be a lesson for countries and regions other than high-SDI ones, which may also be helpful for antibiotic resistance control. Detail methods used in high-SDI countries may not be a one-size-fits-all solution, especially in regions under higher pressure from other infectious pathogens. Policymakers in different regions should find a balance among the risks of CDI, antibiotic resistance, and infectious disease treatments [32,33]. Second, as a faecal-oral-transmit pathogen, good hand hygiene with soap and water, disposable medical devices, regular disinfection in health care staff, and patients’ isolation should be basic requirements and recommendations for health faculty [34]. Third, new effective treatment development is commonplace in defending bacterium. Fidaxomicin, with the market name Dificid developed by Optimer (New Jersey, USA) has been approved by the Food and Drug Administration (FDA) since 2011. As a narrow spectrum macrolide, fidaxomicin could target Clostridium difficile nearly without disrupting normal gut bacterium. The exemplary performance in CDI treatment has made fidaxomicin the first-line recommendation along with vancomycin for initial and recurrent CDI. Although a detailed mechanism has not been identified, faecal microbiota transplantation (FMT) is a safe method to re-establish microflora, assisting in the treatment of CDI. So far, the vaccination and antibody for CDI are still under-estimated in clinical trials [33]. Novel treatment development and lowering their prices could be a guiding principle for health workers and policy makers all around the world.

There are some limitations in our analysis. First, although our study provided an important reference for CDI’s 30-year global trends, it could not elucidate the patterns and impacts of CDI across various timeframes and geographical areas in the context of the COVID-19 pandemic due to the lack of detailed global surveillance, antibiotic consumption, and adverse events reporting data from 2020–2024. It is urgent to articulate the impact of COVID-19 on CDI in future studies once public data are uncovered a few years later. Second, the aforementioned lack of available global data for HA-CDI, CA-CDI, epidemic strains, low-SDI regions’ surveillance data, and the consumption of fidaxomicin and FMT impedes further analysis to propagate uncertainty for some covariates throughout our analytical process. We are calling for more input data by establishing a planet hospital-community CDI circulation and epidemic ribotype surveillance system, improving the newly developed African and Oceanian countries’ surveillance systems, and expecting fidaxomicin and FMT market data to be unveiled in the next 10 years. Third, because our analysis is based on GBD 2019, the Antibiotic map, FAERS, and GLASS databases, it will share some overall limitations and biases from respective data sets. For example, the lags in data availability, retrospective data collection methods, and coding variation from GBD 2019 would always be brought up for global disease trends estimation, because the primary constraint in the GBD analysis of disease burden lies in the accessibility of primary data. The out-of-sample prediction, case definition or measurement method, and collinearity between covariates may influence the ability to capture uncertainty. For FAERS, the heterogeneous reporting origins from both non-health care and health care practitioners are the best-known bias, because reporting may be influenced by nomenclature differences, selection bias, and under-reporting. Furthermore, the lack of detailed clinical information impedes us from judging other covariates such as previous treatment regimens. The main limitations noted from the antibiotic-map and GLASS would be incomplete data, population (DDD methodology not suitable for children), and informal market coverage. Fourth, when analysing epidemiological trends from a global perspective, we often only grasp the general correlations and trend directions, and it's challenging to make explicit inferences, analyses, and explanations of causal relationships. Although we use an approach similar to the fishbone analysis method, focusing on antibiotics as the main cause after excluding some confounding factors, and incorporating FAERS results which provide slightly higher causative evidence than correlation analysis, the reports cannot confirm the causality of the drug-induced event. In the future, more data suitable for causal inference will need to be collected for further analysis.

## CONCLUSIONS

Our study analysed the global burden and trends of Clostridioides difficile-associated deaths and DALYs from 1990 to 2019. ASDRs and ASRDs of Clostridioides difficile increased dramatically in the past 30 years, putting a great burden on high-SDI regions and some high-middle, middle-regions, such as North America, Andean Latin America, and Southern Latin America. Clostridioides difficile stands as the leading cause of antibiotic-associated diarrhoea and one of the most common health care-associated infections, putting a higher burden on females and individuals over 67.5 years old. In-country analysis showed that antibiotic consumption has a significant positive correlation with ASDRs and ASRDs attributed to Clostridioides difficile. Clindamycin, cephalosporins, carbapenems, and penicillin are high-risk antibiotics, while fluoroquinolones, macrolides, and tetracyclines are moderate-risk ones. Strategies used in high-SDI regions are worthy of emulation for others such as targeted interventions in high-risk areas, strategies for antibiotic stewardship, and innovations in CDI prevention and treatment.

## Additional material


Online Supplementary Document
