# Supplementary material for: The global burden and trend of Clostridioides difficile and its association with world antibiotic consumption, 1990–2019
Source: J Glob Health. 2024 Aug 16;14:04135. doi: 10.7189/jogh.14.04135 (PMC11327847; doi:10.7189/jogh.14.04135)
Supplement: Online Supplementary Document [file jogh-14-04135-s001.pdf]

Methods S1: Global Burden Data Source and GBD Estimation

Data on the global burden of CDI were obtained from the Global Burden of Disease Study 2019 using the open online GHDx query tool [1]. Informed consent for accessing the GBD data was waived by the University of Washington Institutional Review Board [2]. The number of deaths, death rates, age-standardized death rates (ASDRs), disability-adjusted life of years (DALYs), age-standardized rates of DALYs (ASRDs) of CDI with 95% uncertainty interval (UI) or 95% confidence intervals (CI) from 1990 to 2019 according to sex, age stratification and different locations were obtained for further analysis. UI is a Bayes regression-based parameter considering the differences between calculation methods and the uncertainty of multivariate imputation by chained equations in different countries. UI's results were obtained by repeated sampling through the correlation matrix and would be more often used to describe the outcomes from GBD 2019. CI is calculated from standard error for other outcomes' depiction. The difference between the two is more like a philosophical molecular preference than a mathematical problem [3-4]. DALYs are the cumulative number of years of life lost due to premature mortality (YLLs) and the years lived with a disability (YLDs), representing the overall burden of mortality and nonfatal health consequences. The Socio-Demographic Index (SDI), as an index based on income, education, and fertility level, is a comprehensive indication of regional development status. General methods of GBD 2019 were reported in detail on its website (http://www.healthdata.org/gbd/2019).

Methods S2: Antibiotic Consumption and Adverse Event Data Source and Calculation

The global antibiotic consumption data were obtained from ResistanceMap-AntibiocUse developed by OneHealthTrust (Center for Disease Dynamics, Economics & Policy) [5]. Data on antibiotics sold in retail and hospital pharmacies for all countries or regions were retrieved from the IQVIA MIDAS database. In order to analyze the correlation between antibiotic usage and CDI, the number of defined daily doses (DDDs) is our chosen methodology. DDDs represent the average maintenance dose of an antimicrobial substance per day for its primary use in adults with an associated ATC code. Typically, DDDs for antibiotics are determined based on the treatment of moderately severe infections. Data on antibiotic sales were converted into DDDs using the Anatomical Therapeutic Chemical Classification System developed by the WHO Collaborating Centre for Drug Statistics Methodology. DDDs were further converted to DDDs per 1,000 inhabitants based on population size provided by the World Bank's database for cross-regional comparison. This approach allows us to use DDDs as a recognized indicator by the World Health Organization to consider various factors such as types of antibiotics, duration of drug use, dosage, length of hospital stay, infectious disease tracking, and complications. By utilizing this method, we can offer a global perspective on antibiotic usage, as detailed in our publication. The antibiotic adverse event data were collected from the publicly available FAERS database from

2004 to 2024. FAERS is a database containing drug-associated post-market adverse events reported by manufacturers, patients, or healthcare professionals at the point of care [6]. Preferred terms identified for Clostridioides difficile from the Medical Dictionary for Regulatory Activities are “Clostridium difficile” “Clostridioides difficile” “Clostridium difficile colitis” “Clostridioides difficile colitis”, “Clostridium colitis”, “Pseudomembranous colitis” and “Clostridioides difficile infection”. Only antibiotics reported as “PS’ (Primary Suspect Drug) were included in further analysis.

Methods S3: Jointpoint Regression Model Analysis

The Jointpoint regression, as called change point regression, was applied to access annual percentage changes (APCs) and average annual percentage changes (AAPCs) in time series data (ASDR, ASRD) [7]. The tests of significance use a Monte Carlo Permutation method. AAPCs significantly differing from 0 in the Z test would be regarded as statistically significant increasing or decreasing trends from 1990 to 2019.

Methods S4: Age-Period-Cohort Analysis

Age-period-cohort models were used to estimate period, age, and cohort effects in temporal trends, referring to the differences across age groups, birth years, and other people-related risk factors [8]. Age and period were divided into 5-year continuous intervals from 5–9 to 90–94, and from 1990–1994 to 2015–2019, respectively.

Twenty-three birth cohorts were summarized from 1985–1904 to 2005–2014. Relative risks (RR) were calculated based on the estimated coefficients to quantify the effects of age, period, and cohort. Wald chi-squared tests were applied to estimate the statistical significance.

Methods S5: Statistical Analysis

Estimated annual percentage changes (EAPCs) were manually calculated to measure the annual trend changes in death and DALY rates. Spearman correlation coefficients (ρ) were calculated to evaluate the relationship between different antibiotic consumption and ASDRs. The Reporting Odds Ratios (ROR) were calculated to estimate the association between CDI and individual antibiotics. P value <0.05 was considered statistically significant. All statistical analyses were performed by R software (version 4.3.1).

Table S1. The rank of the annual rate of changes in 13 pathogens causing diarrheal deaths

Rank	Pathogen	Location	Age	Sex	Year	Annual rate of change
1	Clostridium difficile	Global	All	Both	1990-2019	641.2 (415.4-915.1)
2	Cholera	Global	All	Both	1990-2019	170.5 (54.1-365.6)
3	Enterotoxigenic E coli	Global	All	Both	1990-2019	5.9 (-14.29-29.43)
4	Norovirus	Global	All	Both	1990-2019	5.5 (-24.0-23.8)
5	Campylobacter	Global	All	Both	1990-2019	4.3 (-21.3-26.0)
6	Cryptosporidium	Global	All	Both	1990-2019	-2.6 (-20.1-12.7)
7	Shigella	Global	All	Both	1990-2019	-6.1 (-23.0-12.5)
8	Aeromonas	Global	All	Both	1990-2019	-9.8 (-29.8-9.8)
9	Non-typhoid Salmonella	Global	All	Both	1990-2019	-13.5 (-54.8-12.4)
10	Adenovirus	Global	All	Both	1990-2019	-14.2 (-34.2-6.5)
11	Enteropathogenic E coli	Global	All	Both	1990-2019	-18.9 (-37.2-0.7)
12	Entamoeba	Global	All	Both	1990-2019	-26.4 (-40.4--12.3)
13	Rotavirus	Global	All	Both	1990-2019	-31.9 (-44.5--19.8)

Table S2. The rank of the annual rate of changes in 13 pathogens causing diarrheal DALYs

Rank	Pathogen	Location	Age	Sex	Year	Annual rate of change
1	Clostridium difficile	Global	All	Both	1990-2019	1.03 (0.69-1.42)
2	Cholera	Global	All	Both	1990-2019	0.34 (-0.17-1.08)
3	Shigella	Global	All	Both	1990-2019	-0.55(-0.66--0.37)
4	Campylobacter	Global	All	Both	1990-2019	-0.56 (-0.67--0.39)
5	Norovirus	Global	All	Both	1990-2019	-0.56 (-0.67--0.39)
6	Aeromonas	Global	All	Both	1990-2019	-0.56 (-0.67--0.40)
7	Cryptosporidium	Global	All	Both	1990-2019	-0.58 (-0.68--0.42)
8	Adenovirus	Global	All	Both	1990-2019	-0.58 (-0.70--0.39)
9	Enteropathogenic E coli	Global	All	Both	1990-2019	-0.59 (-0.69--0.44)
10	Non-typhoid Salmonella	Global	All	Both	1990-2019	-0.60 (-0.74--0.40)
11	Enterotoxigenic E coli	Global	All	Both	1990-2019	-0.62 (-0.71--0.49)
12	Entamoeba	Global	All	Both	1990-2019	-0.64(-0.72--0.51)
13	Rotavirus	Global	All	Both	1990-2019	-0.68 (-0.75--0.57)

Table S3. The annual change of rate in Clostridium difficile caused ASDR from 1990 to 2019 in different countries or territories, or other area of its authorities.

measure	location	sex	age	cause	rei	metric	year_start	year_end	val	upper	lower
Deaths	Argentina	Both	Age-standardized	Diarrheal diseases	Clostridium difficile	Rate	1990	2019	29.74641	59.29638	13.97942
Deaths	Paraguay	Both	Age-standardized	Diarrheal diseases	Clostridium difficile	Rate	1990	2019	21.85319	49.00471	10.34788
Deaths	Uruguay	Both	Age-standardized	Diarrheal diseases	Clostridium difficile	Rate	1990	2019	18.89482	31.10526	11.57743
Deaths	Equatorial Guinea	Both	Age-standardized	Diarrheal diseases	Clostridium difficile	Rate	1990	2019	17.0848	36.58435	10.01548
Deaths	Austria	Both	Age-standardized	Diarrheal diseases	Clostridium difficile	Rate	1990	2019	13.06999	15.76684	10.7917
Deaths	Colombia	Both	Age-standardized	Diarrheal diseases	Clostridium difficile	Rate	1990	2019	12.61879	24.14921	6.590914
Deaths	Venezuela (Bolivarian Republic of)	Both	Age-standardized	Diarrheal diseases	Clostridium difficile	Rate	1990	2019	10.66039	16.93128	7.134677
Deaths	Chile	Both	Age-standardized	Diarrheal diseases	Clostridium difficile	Rate	1990	2019	10.24435	16.12417	6.512217
Deaths	Saudi Arabia	Both	Age-standardized	Diarrheal diseases	Clostridium difficile	Rate	1990	2019	9.195094	20.33742	4.68038
Deaths	Saint Lucia	Both	Age-standardized	Diarrheal diseases	Clostridium difficile	Rate	1990	2019	9.132477	18.94655	4.80211
Deaths	Peru	Both	Age-standardized	Diarrheal diseases	Clostridium difficile	Rate	1990	2019	9.132445	16.39191	5.729421
Deaths	Grenada	Both	Age-standardized	Diarrheal diseases	Clostridium difficile	Rate	1990	2019	9.0313	19.46947	4.248953
Deaths	Oman	Both	Age-standardized	Diarrheal diseases	Clostridium difficile	Rate	1990	2019	9.004753	18.91961	4.520693
Deaths	Ecuador	Both	Age-standardized	Diarrheal diseases	Clostridium difficile	Rate	1990	2019	8.601527	15.10185	5.227404
Deaths	Cabo Verde	Both	Age-standardized	Diarrheal diseases	Clostridium difficile	Rate	1990	2019	7.842655	13.91102	4.752514
Deaths	Bolivia (Plurinational State of)	Both	Age-standardized	Diarrheal diseases	Clostridium difficile	Rate	1990	2019	7.755334	14.94192	4.245542
Deaths	Panama	Both	Age-standardized	Diarrheal diseases	Clostridium difficile	Rate	1990	2019	7.735773	11.88127	5.356872

Deaths	El Salvador	Both	Age-standardized	Diarrheal diseases	Clostridium difficile	Rate	1990	2019	7.702155	17.93096	3.862403
Deaths	United States of America	Both	Age-standardized	Diarrheal diseases	Clostridium difficile	Rate	1990	2019	7.358705	8.167635	6.544842
Deaths	Portugal	Both	Age-standardized	Diarrheal diseases	Clostridium difficile	Rate	1990	2019	7.289317	10.81004	5.334962
Deaths	Jamaica	Both	Age-standardized	Diarrheal diseases	Clostridium difficile	Rate	1990	2019	5.564477	11.58036	2.840987
Deaths	Canada	Both	Age-standardized	Diarrheal diseases	Clostridium difficile	Rate	1990	2019	5.420375	6.689413	4.296289
Deaths	Costa Rica	Both	Age-standardized	Diarrheal diseases	Clostridium difficile	Rate	1990	2019	5.402068	9.123259	3.17829
Deaths	Saint Vincent and the Grenadines	Both	Age-standardized	Diarrheal diseases	Clostridium difficile	Rate	1990	2019	5.124411	10.32052	2.863674
Deaths	Dominica	Both	Age-standardized	Diarrheal diseases	Clostridium difficile	Rate	1990	2019	4.990616	9.738978	2.441712
Deaths	Suriname	Both	Age-standardized	Diarrheal diseases	Clostridium difficile	Rate	1990	2019	4.416814	8.696337	2.639005
Deaths	Honduras	Both	Age-standardized	Diarrheal diseases	Clostridium difficile	Rate	1990	2019	4.107428	9.078098	1.780165
Deaths	United Kingdom	Both	Age-standardized	Diarrheal diseases	Clostridium difficile	Rate	1990	2019	3.972654	4.627304	3.344149
Deaths	Belize	Both	Age-standardized	Diarrheal diseases	Clostridium difficile	Rate	1990	2019	3.867464	8.227413	1.89985
Deaths	Turkey	Both	Age-standardized	Diarrheal diseases	Clostridium difficile	Rate	1990	2019	3.655573	8.193984	1.585172
Deaths	Hungary	Both	Age-standardized	Diarrheal diseases	Clostridium difficile	Rate	1990	2019	3.548445	4.479304	2.791857
Deaths	Maldives	Both	Age-standardized	Diarrheal diseases	Clostridium difficile	Rate	1990	2019	3.491666	7.383918	1.647515
Deaths	Sweden	Both	Age-standardized	Diarrheal diseases	Clostridium difficile	Rate	1990	2019	3.465762	4.486246	2.558104
Deaths	Guatemala	Both	Age-standardized	Diarrheal diseases	Clostridium difficile	Rate	1990	2019	3.298011	7.961883	1.439678
Deaths	Bhutan	Both	Age-standardized	Diarrheal diseases	Clostridium difficile	Rate	1990	2019	3.240356	5.429703	2.262566
Deaths	Nicaragua	Both	Age-standar	Diarrheal	Clostridium	Rate	1990	2019	3.120848	7.064645	1.332105

			dized	diseases	difficile						
Deaths	Romania	Both	Age-standardized	Diarrheal diseases	Clostridium difficile	Rate	1990	2019	2.897301	6.884251	1.235582
Deaths	North Macedonia	Both	Age-standardized	Diarrheal diseases	Clostridium difficile	Rate	1990	2019	2.871553	6.705158	1.175011
Deaths	Iran (Islamic Republic of)	Both	Age-standardized	Diarrheal diseases	Clostridium difficile	Rate	1990	2019	2.72563	4.659984	1.493124
Deaths	Bosnia and Herzegovina	Both	Age-standardized	Diarrheal diseases	Clostridium difficile	Rate	1990	2019	2.706038	6.230817	1.141388
Deaths	Ireland	Both	Age-standardized	Diarrheal diseases	Clostridium difficile	Rate	1990	2019	2.652806	3.466348	2.008941
Deaths	Netherlands	Both	Age-standardized	Diarrheal diseases	Clostridium difficile	Rate	1990	2019	2.360709	3.147519	1.784485
Deaths	Antigua and Barbuda	Both	Age-standardized	Diarrheal diseases	Clostridium difficile	Rate	1990	2019	2.326104	3.648917	1.410869
Deaths	Guyana	Both	Age-standardized	Diarrheal diseases	Clostridium difficile	Rate	1990	2019	2.288048	4.908226	1.05238
Deaths	Mexico	Both	Age-standardized	Diarrheal diseases	Clostridium difficile	Rate	1990	2019	2.276295	3.542604	1.353832
Deaths	Dominican Republic	Both	Age-standardized	Diarrheal diseases	Clostridium difficile	Rate	1990	2019	2.131659	4.639894	0.926974
Deaths	Trinidad and Tobago	Both	Age-standardized	Diarrheal diseases	Clostridium difficile	Rate	1990	2019	2.070992	3.400165	0.993261
Deaths	Italy	Both	Age-standardized	Diarrheal diseases	Clostridium difficile	Rate	1990	2019	2.052392	2.401736	1.687284
Deaths	Germany	Both	Age-standardized	Diarrheal diseases	Clostridium difficile	Rate	1990	2019	2.051413	2.518135	1.627094
Deaths	Algeria	Both	Age-standardized	Diarrheal diseases	Clostridium difficile	Rate	1990	2019	2.045727	4.147344	0.971945
Deaths	Israel	Both	Age-standardized	Diarrheal diseases	Clostridium difficile	Rate	1990	2019	2.035161	2.522836	1.582718
Deaths	Belgium	Both	Age-standardized	Diarrheal diseases	Clostridium difficile	Rate	1990	2019	2.015463	2.481891	1.601127
Deaths	Sao Tome and Principe	Both	Age-standardized	Diarrheal diseases	Clostridium difficile	Rate	1990	2019	1.978732	3.465277	1.110028
Deaths	Czechia	Both	Age-standardized	Diarrheal diseases	Clostridium difficile	Rate	1990	2019	1.809453	2.3083	1.389001

Deaths	Tunisia	Both	Age-standardized	Diarrheal diseases	Clostridium difficile	Rate	1990	2019	1.759186	3.798069	0.883679
Deaths	Poland	Both	Age-standardized	Diarrheal diseases	Clostridium difficile	Rate	1990	2019	1.704506	2.749043	0.948449
Deaths	Libya	Both	Age-standardized	Diarrheal diseases	Clostridium difficile	Rate	1990	2019	1.620471	3.113252	0.711317
Deaths	Syrian Arab Republic	Both	Age-standardized	Diarrheal diseases	Clostridium difficile	Rate	1990	2019	1.603904	3.523062	0.643555
Deaths	Jordan	Both	Age-standardized	Diarrheal diseases	Clostridium difficile	Rate	1990	2019	1.581297	3.362096	0.631198
Deaths	Viet Nam	Both	Age-standardized	Diarrheal diseases	Clostridium difficile	Rate	1990	2019	1.57485	3.049247	0.725733
Deaths	Lebanon	Both	Age-standardized	Diarrheal diseases	Clostridium difficile	Rate	1990	2019	1.563862	3.157769	0.717526
Deaths	Norway	Both	Age-standardized	Diarrheal diseases	Clostridium difficile	Rate	1990	2019	1.431027	1.735318	1.133647
Deaths	Uzbekistan	Both	Age-standardized	Diarrheal diseases	Clostridium difficile	Rate	1990	2019	1.426431	3.108425	0.573679
Deaths	Republic of Korea	Both	Age-standardized	Diarrheal diseases	Clostridium difficile	Rate	1990	2019	1.384483	2.207904	0.714977
Deaths	Saint Kitts and Nevis	Both	Age-standardized	Diarrheal diseases	Clostridium difficile	Rate	1990	2019	1.379422	2.35356	0.616353
Deaths	Australia	Both	Age-standardized	Diarrheal diseases	Clostridium difficile	Rate	1990	2019	1.367447	1.758564	0.980766
Deaths	Greenland	Both	Age-standardized	Diarrheal diseases	Clostridium difficile	Rate	1990	2019	1.281585	2.320492	0.342109
Deaths	Kazakhstan	Both	Age-standardized	Diarrheal diseases	Clostridium difficile	Rate	1990	2019	1.242528	3.164693	0.326717
Deaths	Denmark	Both	Age-standardized	Diarrheal diseases	Clostridium difficile	Rate	1990	2019	1.149814	1.491113	0.863378
Deaths	Albania	Both	Age-standardized	Diarrheal diseases	Clostridium difficile	Rate	1990	2019	1.137699	3.098146	0.285102
Deaths	Greece	Both	Age-standardized	Diarrheal diseases	Clostridium difficile	Rate	1990	2019	1.13154	1.593211	0.774981
Deaths	New Zealand	Both	Age-standardized	Diarrheal diseases	Clostridium difficile	Rate	1990	2019	1.100107	1.549157	0.743029
Deaths	Botswana	Both	Age-standar	Diarrheal	Clostridium	Rate	1990	2019	1.09896	1.543785	0.761837

			dized	diseases	difficile						
Deaths	Brunei Darussalam	Both	Age-standardized	Diarrheal diseases	Clostridium difficile	Rate	1990	2019	1.083412	1.500092	0.660917
Deaths	Cuba	Both	Age-standardized	Diarrheal diseases	Clostridium difficile	Rate	1990	2019	1.083134	1.86899	0.52898
Deaths	Iceland	Both	Age-standardized	Diarrheal diseases	Clostridium difficile	Rate	1990	2019	1.072533	1.475141	0.742101
Deaths	Puerto Rico	Both	Age-standardized	Diarrheal diseases	Clostridium difficile	Rate	1990	2019	1.064902	1.380046	0.799244
Deaths	Armenia	Both	Age-standardized	Diarrheal diseases	Clostridium difficile	Rate	1990	2019	1.057867	2.190659	0.351184
Deaths	Angola	Both	Age-standardized	Diarrheal diseases	Clostridium difficile	Rate	1990	2019	1.004884	2.417398	0.305981
Deaths	Spain	Both	Age-standardized	Diarrheal diseases	Clostridium difficile	Rate	1990	2019	0.96574	1.262369	0.715956
Deaths	Turkmenistan	Both	Age-standardized	Diarrheal diseases	Clostridium difficile	Rate	1990	2019	0.880583	2.031571	0.263605
Deaths	Malta	Both	Age-standardized	Diarrheal diseases	Clostridium difficile	Rate	1990	2019	0.868165	1.311338	0.488738
Deaths	Azerbaijan	Both	Age-standardized	Diarrheal diseases	Clostridium difficile	Rate	1990	2019	0.861499	1.97796	0.36257
Deaths	Gabon	Both	Age-standardized	Diarrheal diseases	Clostridium difficile	Rate	1990	2019	0.859972	1.220695	0.548002
Deaths	Serbia	Both	Age-standardized	Diarrheal diseases	Clostridium difficile	Rate	1990	2019	0.798588	1.697202	0.142701
Deaths	Luxembourg	Both	Age-standardized	Diarrheal diseases	Clostridium difficile	Rate	1990	2019	0.793749	1.090926	0.5184
Deaths	Sri Lanka	Both	Age-standardized	Diarrheal diseases	Clostridium difficile	Rate	1990	2019	0.737436	1.194803	0.268388
Deaths	China	Both	Age-standardized	Diarrheal diseases	Clostridium difficile	Rate	1990	2019	0.735771	1.576372	0.170909
Deaths	Mauritania	Both	Age-standardized	Diarrheal diseases	Clostridium difficile	Rate	1990	2019	0.731901	1.309038	0.385254
Deaths	Bulgaria	Both	Age-standardized	Diarrheal diseases	Clostridium difficile	Rate	1990	2019	0.658702	1.190003	0.249298
Deaths	Myanmar	Both	Age-standardized	Diarrheal diseases	Clostridium difficile	Rate	1990	2019	0.657517	1.738716	0.106373

Deaths	Russian Federation	Both	Age-standardized	Diarrheal diseases	Clostridium difficile	Rate	1990	2019	0.590754	1.281332	0.148215
Deaths	Belarus	Both	Age-standardized	Diarrheal diseases	Clostridium difficile	Rate	1990	2019	0.57699	1.991978	-0.01547
Deaths	Slovakia	Both	Age-standardized	Diarrheal diseases	Clostridium difficile	Rate	1990	2019	0.566677	1.118631	0.112613
Deaths	Qatar	Both	Age-standardized	Diarrheal diseases	Clostridium difficile	Rate	1990	2019	0.555226	1.150692	0.08558
Deaths	Lithuania	Both	Age-standardized	Diarrheal diseases	Clostridium difficile	Rate	1990	2019	0.50426	0.947252	0.136781
Deaths	Switzerland	Both	Age-standardized	Diarrheal diseases	Clostridium difficile	Rate	1990	2019	0.495797	0.741062	0.279541
Deaths	Cyprus	Both	Age-standardized	Diarrheal diseases	Clostridium difficile	Rate	1990	2019	0.488218	1.051514	-0.05458
Deaths	Mozambique	Both	Age-standardized	Diarrheal diseases	Clostridium difficile	Rate	1990	2019	0.466809	1.235817	-0.01898
Deaths	Singapore	Both	Age-standardized	Diarrheal diseases	Clostridium difficile	Rate	1990	2019	0.450291	0.911097	0.092235
Deaths	Barbados	Both	Age-standardized	Diarrheal diseases	Clostridium difficile	Rate	1990	2019	0.438085	0.868144	0.125449
Deaths	Indonesia	Both	Age-standardized	Diarrheal diseases	Clostridium difficile	Rate	1990	2019	0.420034	0.803234	0.143414
Deaths	Bahamas	Both	Age-standardized	Diarrheal diseases	Clostridium difficile	Rate	1990	2019	0.415656	0.772296	0.101993
Deaths	Uganda	Both	Age-standardized	Diarrheal diseases	Clostridium difficile	Rate	1990	2019	0.409464	1.062358	0.038043
Deaths	Cambodia	Both	Age-standardized	Diarrheal diseases	Clostridium difficile	Rate	1990	2019	0.406007	1.257007	-0.03024
Deaths	Ghana	Both	Age-standardized	Diarrheal diseases	Clostridium difficile	Rate	1990	2019	0.402774	0.61918	0.219111
Deaths	Monaco	Both	Age-standardized	Diarrheal diseases	Clostridium difficile	Rate	1990	2019	0.386417	1.063161	-0.11708
Deaths	United Arab Emirates	Both	Age-standardized	Diarrheal diseases	Clostridium difficile	Rate	1990	2019	0.377893	0.763972	-0.02716
Deaths	Yemen	Both	Age-standardized	Diarrheal diseases	Clostridium difficile	Rate	1990	2019	0.360059	0.881084	0.061806
Deaths	Japan	Both	Age-standar	Diarrheal	Clostridium	Rate	1990	2019	0.359418	0.498303	0.232862

			dized	disea ses	difficile						
Deat hs	France	Bo th	Age-standar dized	Diarrh eal disea ses	Clostri dium difficile	Rat e	1990	2019	0.307 223	0.619 861	0.061 891
Deat hs	Montenegro	Bo th	Age-standar dized	Diarrh eal disea ses	Clostri dium difficile	Rat e	1990	2019	0.303 186	0.753 945	- 0.066 73
Deat hs	Malawi	Bo th	Age-standar dized	Diarrh eal disea ses	Clostri dium difficile	Rat e	1990	2019	0.295 671	0.889 191	- 0.047 19
Deat hs	Rwanda	Bo th	Age-standar dized	Diarrh eal disea ses	Clostri dium difficile	Rat e	1990	2019	0.284 998	0.817 972	- 0.080 77
Deat hs	United Republic of Tanzania	Bo th	Age-standar dized	Diarrh eal disea ses	Clostri dium difficile	Rat e	1990	2019	0.273 457	0.731 625	- 0.029 73
Deat hs	Eritrea	Bo th	Age-standar dized	Diarrh eal disea ses	Clostri dium difficile	Rat e	1990	2019	0.259 66	1.145 247	- 0.131 31
Deat hs	Nepal	Bo th	Age-standar dized	Diarrh eal disea ses	Clostri dium difficile	Rat e	1990	2019	0.250 827	0.785 179	- 0.111 1
Deat hs	Haiti	Bo th	Age-standar dized	Diarrh eal disea ses	Clostri dium difficile	Rat e	1990	2019	0.241 891	0.783 512	- 0.180 83
Deat hs	Egypt	Bo th	Age-standar dized	Diarrh eal disea ses	Clostri dium difficile	Rat e	1990	2019	0.217 824	1.003 436	- 0.256 12
Deat hs	India	Bo th	Age-standar dized	Diarrh eal disea ses	Clostri dium difficile	Rat e	1990	2019	0.215 638	0.470 39	0.027 223
Deat hs	Croatia	Bo th	Age-standar dized	Diarrh eal disea ses	Clostri dium difficile	Rat e	1990	2019	0.209 021	0.485 522	- 0.038 52
Deat hs	Kenya	Bo th	Age-standar dized	Diarrh eal disea ses	Clostri dium difficile	Rat e	1990	2019	0.191 959	0.596 713	- 0.095 2
Deat hs	Morocco	Bo th	Age-standar dized	Diarrh eal disea ses	Clostri dium difficile	Rat e	1990	2019	0.190 473	0.951 327	- 0.273 98
Deat hs	Sudan	Bo th	Age-standar dized	Diarrh eal disea ses	Clostri dium difficile	Rat e	1990	2019	0.184 236	0.597 541	- 0.092 16
Deat hs	Iraq	Bo th	Age-standar dized	Diarrh eal disea ses	Clostri dium difficile	Rat e	1990	2019	0.174 097	0.675 408	- 0.148 36
Deat hs	Djibouti	Bo th	Age-standar dized	Diarrh eal disea ses	Clostri dium difficile	Rat e	1990	2019	0.173 485	0.344 261	0.041 414
Deat hs	Comoros	Bo th	Age-standar dized	Diarrh eal disea ses	Clostri dium difficile	Rat e	1990	2019	0.169 572	0.390 511	- 0.016 3
Deat hs	Estonia	Bo th	Age-standar dized	Diarrh eal disea ses	Clostri dium difficile	Rat e	1990	2019	0.157 514	0.533 592	- 0.119 55

Deaths	Palestine	Both	Age-standardized	Diarrheal diseases	Clostridium difficile	Rate	1990	2019	0.153072	0.758733	-0.17465
Deaths	San Marino	Both	Age-standardized	Diarrheal diseases	Clostridium difficile	Rate	1990	2019	0.131446	0.743209	-0.26521
Deaths	Namibia	Both	Age-standardized	Diarrheal diseases	Clostridium difficile	Rate	1990	2019	0.127459	0.263719	0.012363
Deaths	Republic of Moldova	Both	Age-standardized	Diarrheal diseases	Clostridium difficile	Rate	1990	2019	0.120954	1.082771	-0.29367
Deaths	United States Virgin Islands	Both	Age-standardized	Diarrheal diseases	Clostridium difficile	Rate	1990	2019	0.097615	0.41065	-0.17556
Deaths	South Africa	Both	Age-standardized	Diarrheal diseases	Clostridium difficile	Rate	1990	2019	0.096696	0.297879	-0.0732
Deaths	Ethiopia	Both	Age-standardized	Diarrheal diseases	Clostridium difficile	Rate	1990	2019	0.081486	0.361282	-0.1376
Deaths	Lao People's Democratic Republic	Both	Age-standardized	Diarrheal diseases	Clostridium difficile	Rate	1990	2019	0.073138	0.555589	-0.27511
Deaths	Afghanistan	Both	Age-standardized	Diarrheal diseases	Clostridium difficile	Rate	1990	2019	0.05115	0.41321	-0.19937
Deaths	Senegal	Both	Age-standardized	Diarrheal diseases	Clostridium difficile	Rate	1990	2019	0.036877	0.225046	-0.15345
Deaths	Mongolia	Both	Age-standardized	Diarrheal diseases	Clostridium difficile	Rate	1990	2019	0.034497	0.482818	-0.27277
Deaths	Togo	Both	Age-standardized	Diarrheal diseases	Clostridium difficile	Rate	1990	2019	0.026136	0.241189	-0.16799
Deaths	Tajikistan	Both	Age-standardized	Diarrheal diseases	Clostridium difficile	Rate	1990	2019	0.014487	0.499397	-0.23421
Deaths	Andorra	Both	Age-standardized	Diarrheal diseases	Clostridium difficile	Rate	1990	2019	-0.00975	0.3732	-0.30646
Deaths	Niger	Both	Age-standardized	Diarrheal diseases	Clostridium difficile	Rate	1990	2019	-0.02046	0.463984	-0.30851
Deaths	Tokelau	Both	Age-standardized	Diarrheal diseases	Clostridium difficile	Rate	1990	2019	-0.02138	0.139281	-0.20766
Deaths	Bangladesh	Both	Age-standardized	Diarrheal diseases	Clostridium difficile	Rate	1990	2019	-0.02298	0.312202	-0.30738
Deaths	Burkina Faso	Both	Age-standardized	Diarrheal diseases	Clostridium difficile	Rate	1990	2019	-0.02869	0.375711	-0.33254
Deaths	Tuvalu	Both	Age-standar	Diarrheal	Clostridium	Rate	1990	2019	-0.037	0.136621	-0.180

			dized	diseases	difficile				86		84
Deaths	Timor-Leste	Both	Age-standardized	Diarrheal diseases	Clostridium difficile	Rate	1990	2019	-0.04332	0.35748	-0.45492
Deaths	Latvia	Both	Age-standardized	Diarrheal diseases	Clostridium difficile	Rate	1990	2019	-0.04952	0.258058	-0.30649
Deaths	Zambia	Both	Age-standardized	Diarrheal diseases	Clostridium difficile	Rate	1990	2019	-0.0611	0.138142	-0.27314
Deaths	Chad	Both	Age-standardized	Diarrheal diseases	Clostridium difficile	Rate	1990	2019	-0.06155	0.441082	-0.35028
Deaths	South Sudan	Both	Age-standardized	Diarrheal diseases	Clostridium difficile	Rate	1990	2019	-0.07538	0.394877	-0.42875
Deaths	Guinea-Bissau	Both	Age-standardized	Diarrheal diseases	Clostridium difficile	Rate	1990	2019	-0.07826	0.31669	-0.37072
Deaths	Cameroon	Both	Age-standardized	Diarrheal diseases	Clostridium difficile	Rate	1990	2019	-0.09664	0.066015	-0.24168
Deaths	Thailand	Both	Age-standardized	Diarrheal diseases	Clostridium difficile	Rate	1990	2019	-0.09717	0.057444	-0.27068
Deaths	Slovenia	Both	Age-standardized	Diarrheal diseases	Clostridium difficile	Rate	1990	2019	-0.10096	0.216909	-0.35497
Deaths	Kyrgyzstan	Both	Age-standardized	Diarrheal diseases	Clostridium difficile	Rate	1990	2019	-0.1117	0.279343	-0.35619
Deaths	Mali	Both	Age-standardized	Diarrheal diseases	Clostridium difficile	Rate	1990	2019	-0.11971	0.345684	-0.39735
Deaths	Papua New Guinea	Both	Age-standardized	Diarrheal diseases	Clostridium difficile	Rate	1990	2019	-0.1245	0.340427	-0.44025
Deaths	Bahrain	Both	Age-standardized	Diarrheal diseases	Clostridium difficile	Rate	1990	2019	-0.12848	0.028375	-0.28797
Deaths	Ukraine	Both	Age-standardized	Diarrheal diseases	Clostridium difficile	Rate	1990	2019	-0.133	0.153519	-0.33418
Deaths	Kiribati	Both	Age-standardized	Diarrheal diseases	Clostridium difficile	Rate	1990	2019	-0.13735	0.286964	-0.46891
Deaths	Congo	Both	Age-standardized	Diarrheal diseases	Clostridium difficile	Rate	1990	2019	-0.15903	-0.02789	-0.29323
Deaths	Micronesia (Federated States of)	Both	Age-standardized	Diarrheal diseases	Clostridium difficile	Rate	1990	2019	-0.16792	-0.01643	-0.52401
Deaths	Madagascar	Both	Age-standardized	Diarrheal diseases	Clostridium difficile	Rate	1990	2019	-0.17146	0.227206	-0.49752

Deat hs	Brazil	Bo th	Age-standar dized	Diarrh eal disea ses	Clostri dium difficile	Rat e	1990	2019	- 0.185 39	0.010 668	- 0.337 97
Deat hs	Guinea	Bo th	Age-standar dized	Diarrh eal disea ses	Clostri dium difficile	Rat e	1990	2019	- 0.186 28	0.090 349	- 0.454 91
Deat hs	Kuwait	Bo th	Age-standar dized	Diarrh eal disea ses	Clostri dium difficile	Rat e	1990	2019	- 0.193 05	0.076 419	- 0.430 56
Deat hs	Sierra Leone	Bo th	Age-standar dized	Diarrh eal disea ses	Clostri dium difficile	Rat e	1990	2019	- 0.197 3	0.146 77	- 0.485 52
Deat hs	Liberia	Bo th	Age-standar dized	Diarrh eal disea ses	Clostri dium difficile	Rat e	1990	2019	- 0.203 99	0.201 988	- 0.536 71
Deat hs	Solomon Islands	Bo th	Age-standar dized	Diarrh eal disea ses	Clostri dium difficile	Rat e	1990	2019	- 0.208 56	0.205 351	- 0.499 74
Deat hs	Gambia	Bo th	Age-standar dized	Diarrh eal disea ses	Clostri dium difficile	Rat e	1990	2019	- 0.268 58	- 0.113 25	- 0.460 52
Deat hs	Lesotho	Bo th	Age-standar dized	Diarrh eal disea ses	Clostri dium difficile	Rat e	1990	2019	- 0.274 33	0.003 779	- 0.526 2
Deat hs	Malaysia	Bo th	Age-standar dized	Diarrh eal disea ses	Clostri dium difficile	Rat e	1990	2019	- 0.295 55	- 0.140 41	- 0.432 64
Deat hs	Benin	Bo th	Age-standar dized	Diarrh eal disea ses	Clostri dium difficile	Rat e	1990	2019	- 0.301 37	- 0.004 74	- 0.560 21
Deat hs	Marshall Islands	Bo th	Age-standar dized	Diarrh eal disea ses	Clostri dium difficile	Rat e	1990	2019	- 0.330 09	- 0.174 05	- 0.503 34
Deat hs	Eswatini	Bo th	Age-standar dized	Diarrh eal disea ses	Clostri dium difficile	Rat e	1990	2019	- 0.336 48	- 0.200 81	- 0.465 34
Deat hs	Somalia	Bo th	Age-standar dized	Diarrh eal disea ses	Clostri dium difficile	Rat e	1990	2019	- 0.344 45	0.012 852	- 0.613 32
Deat hs	Burundi	Bo th	Age-standar dized	Diarrh eal disea ses	Clostri dium difficile	Rat e	1990	2019	- 0.353 47	0.012 733	- 0.650 1
Deat hs	Vanuatu	Bo th	Age-standar dized	Diarrh eal disea ses	Clostri dium difficile	Rat e	1990	2019	- 0.361 17	- 0.057 4	- 0.612 68
Deat hs	Mauritius	Bo th	Age-standar dized	Diarrh eal disea ses	Clostri dium difficile	Rat e	1990	2019	- 0.372 39	- 0.272 57	- 0.462 48
Deat hs	Democratic Republic of the Congo	Bo th	Age-standar dized	Diarrh eal disea ses	Clostri dium difficile	Rat e	1990	2019	- 0.374 68	- 0.009 05	- 0.636 88
Deat hs	Bermuda	Bo th	Age-standar dized	Diarrh eal disea ses	Clostri dium difficile	Rat e	1990	2019	- 0.396 07	- 0.206 58	- 0.536 93
Deat hs	Finland	Bo th	Age-standar	Diarrh eal	Clostri dium	Rat e	1990	2019	- 0.426	- 0.276	- 0.543

			dized	diseases	difficile				17	85	73
Deaths	Central African Republic	Both	Age-standardized	Diarrheal diseases	Clostridium difficile	Rate	1990	2019	-0.44921	-0.14301	-0.69609
Deaths	Taiwan (Province of China)	Both	Age-standardized	Diarrheal diseases	Clostridium difficile	Rate	1990	2019	-0.45212	-0.33595	-0.55617
Deaths	Georgia	Both	Age-standardized	Diarrheal diseases	Clostridium difficile	Rate	1990	2019	-0.4845	-0.36757	-0.60065
Deaths	Tonga	Both	Age-standardized	Diarrheal diseases	Clostridium difficile	Rate	1990	2019	-0.48759	-0.39142	-0.58535
Deaths	C 么 te d'Ivoire	Both	Age-standardized	Diarrheal diseases	Clostridium difficile	Rate	1990	2019	-0.49597	-0.32171	-0.6641
Deaths	Seychelles	Both	Age-standardized	Diarrheal diseases	Clostridium difficile	Rate	1990	2019	-0.5154	-0.43834	-0.5799
Deaths	Guam	Both	Age-standardized	Diarrheal diseases	Clostridium difficile	Rate	1990	2019	-0.5423	-0.43193	-0.62862
Deaths	Palau	Both	Age-standardized	Diarrheal diseases	Clostridium difficile	Rate	1990	2019	-0.54634	-0.45794	-0.62767
Deaths	Nigeria	Both	Age-standardized	Diarrheal diseases	Clostridium difficile	Rate	1990	2019	-0.54634	-0.36372	-0.68196
Deaths	Pakistan	Both	Age-standardized	Diarrheal diseases	Clostridium difficile	Rate	1990	2019	-0.57473	-0.42854	-0.70474
Deaths	Philippines	Both	Age-standardized	Diarrheal diseases	Clostridium difficile	Rate	1990	2019	-0.58517	-0.46821	-0.68392
Deaths	Democratic People's Republic of Korea	Both	Age-standardized	Diarrheal diseases	Clostridium difficile	Rate	1990	2019	-0.61577	-0.43015	-0.76981
Deaths	American Samoa	Both	Age-standardized	Diarrheal diseases	Clostridium difficile	Rate	1990	2019	-0.62934	-0.55158	-0.70312
Deaths	Cook Islands	Both	Age-standardized	Diarrheal diseases	Clostridium difficile	Rate	1990	2019	-0.63866	-0.49667	-0.77464
Deaths	Northern Mariana Islands	Both	Age-standardized	Diarrheal diseases	Clostridium difficile	Rate	1990	2019	-0.65427	-0.55642	-0.73146
Deaths	Niue	Both	Age-standardized	Diarrheal diseases	Clostridium difficile	Rate	1990	2019	-0.65609	-0.57202	-0.72797
Deaths	Nauru	Both	Age-standardized	Diarrheal diseases	Clostridium difficile	Rate	1990	2019	-0.68059	-0.59595	-0.75691
Deaths	Fiji	Both	Age-standardized	Diarrheal diseases	Clostridium difficile	Rate	1990	2019	-0.68124	-0.61251	-0.74578

Deaths	Samoa	Both	Age-standardized	Diarrheal diseases	Clostridium difficile	Rate	1990	2019	-0.78706	-0.71408	-0.84964
Deaths	Zimbabwe	Both	Age-standardized	Diarrheal diseases	Clostridium difficile	Rate	1990	2019	-0.79388	-0.68077	-0.88939

Figures S1. The relationship between unsafe sanitation and lack of essential medical service and CDI burden. ASDR – age-standardized death rate

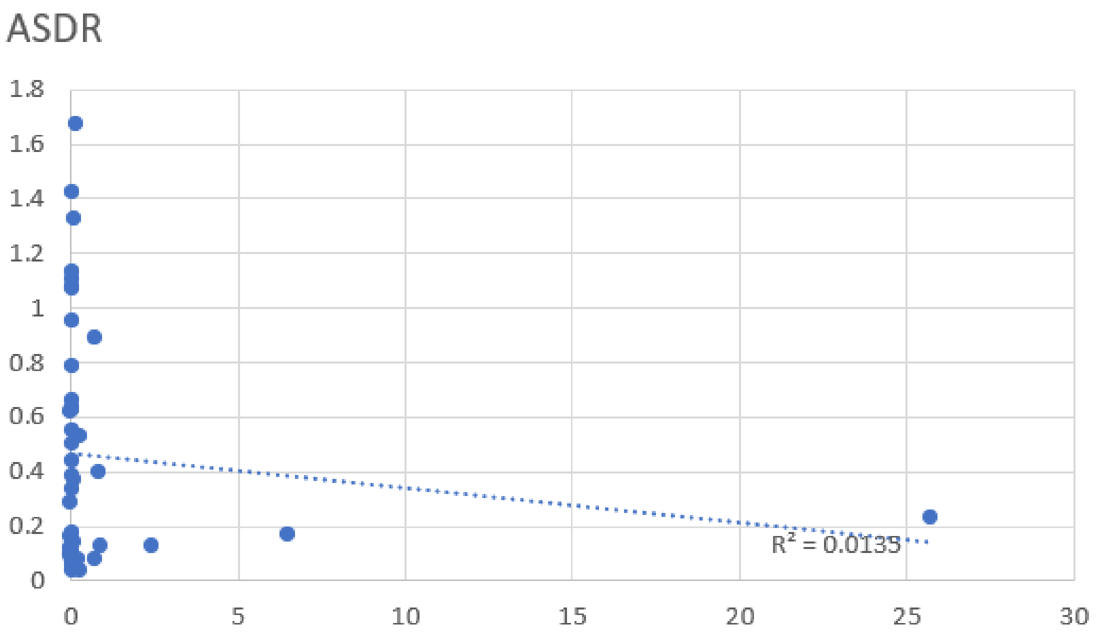

Reference

[1] Global Burden of Disease Collaborative Network. Global Burden of Disease Study 2019 (GBD 2019). Seattle, United States: Institute for Health Metrics and Evaluation (IHME), 2020.

[2] Global burden of 87 risk factors in 204 countries and territories, 1990-2019: a systematic analysis for the Global Burden of Disease Study 2019. Lancet. 2020;396(10258):1223-49.

[3] Gelman A, Greenland S. Are confidence intervals better termed "uncertainty intervals"? BMJ. 2019 Sep 10;366:l5381. doi: 10.1136/bmj.l5381

[4] Cowen S, Ellison SL. Reporting measurement uncertainty and coverage intervals near natural limits. Analyst. 2006 Jun;131(6):710-7. doi: 10.1039/b518084h

[5] The Center for Disease Dynamics, Economics & Policy. ResistanceMap: Antibiotic use. https://resistancemap.OneHealthTrust.org/AntibioticUse.php. Date accessed: Oct 31, 2023. 2023

[6] FDA Adverse Event Reporting System (FAERS). U.S. FOOD & DRUG ADMINISTRATION. https://www.fda.gov/drugs/drug-approvals-and-databases/fda-adverse-event-reporting-system-faers

[7] Kim HJ, Fay MP, Feuer EJ, Midthune DN. Permutation tests for joinpoint regression with applications to cancer rates. Stat Med. 2000;19(3):335-51.

[8] Rosenberg PS, Check DP, Anderson WF. A web tool for age-period-cohort analysis of cancer incidence and mortality rates. Cancer Epidemiol Biomarkers Prev. 2014;23(11):2296-302.
